# Two-particle Bose–Einstein correlations in *pp* collisions at $\mathbf {\sqrt{s} =}$ 0.9 and 7 TeV measured with the ATLAS detector

**DOI:** 10.1140/epjc/s10052-015-3644-x

**Published:** 2015-10-01

**Authors:** G. Aad, B. Abbott, J. Abdallah, S. Abdel Khalek, O. Abdinov, R. Aben, B. Abi, M. Abolins, O. S. AbouZeid, H. Abramowicz, H. Abreu, R. Abreu, Y. Abulaiti, B. S. Acharya, L. Adamczyk, D. L. Adams, J. Adelman, S. Adomeit, T. Adye, T. Agatonovic-Jovin, J. A. Aguilar-Saavedra, M. Agustoni, S. P. Ahlen, F. Ahmadov, G. Aielli, H. Akerstedt, T. P. A. Åkesson, G. Akimoto, A. V. Akimov, G. L. Alberghi, J. Albert, S. Albrand, M. J. Alconada Verzini, M. Aleksa, I. N. Aleksandrov, C. Alexa, G. Alexander, G. Alexandre, T. Alexopoulos, M. Alhroob, G. Alimonti, L. Alio, J. Alison, B. M. M. Allbrooke, L. J. Allison, P. P. Allport, J. Almond, A. Aloisio, A. Alonso, F. Alonso, C. Alpigiani, A. Altheimer, B. Alvarez Gonzalez, M. G. Alviggi, K. Amako, Y. Amaral Coutinho, C. Amelung, D. Amidei, S. P. Amor Dos Santos, A. Amorim, S. Amoroso, N. Amram, G. Amundsen, C. Anastopoulos, L. S. Ancu, N. Andari, T. Andeen, C. F. Anders, G. Anders, K. J. Anderson, A. Andreazza, V. Andrei, X. S. Anduaga, S. Angelidakis, I. Angelozzi, P. Anger, A. Angerami, F. Anghinolfi, A. V. Anisenkov, N. Anjos, A. Annovi, A. Antonaki, M. Antonelli, A. Antonov, J. Antos, F. Anulli, M. Aoki, L. Aperio Bella, R. Apolle, G. Arabidze, I. Aracena, Y. Arai, J. P. Araque, A. T. H. Arce, J-F. Arguin, S. Argyropoulos, M. Arik, A. J. Armbruster, O. Arnaez, V. Arnal, H. Arnold, M. Arratia, O. Arslan, A. Artamonov, G. Artoni, S. Asai, N. Asbah, A. Ashkenazi, B. Åsman, L. Asquith, K. Assamagan, R. Astalos, M. Atkinson, N. B. Atlay, B. Auerbach, K. Augsten, M. Aurousseau, G. Avolio, G. Azuelos, Y. Azuma, M. A. Baak, A. E. Baas, C. Bacci, H. Bachacou, K. Bachas, M. Backes, M. Backhaus, J. Backus Mayes, E. Badescu, P. Bagiacchi, P. Bagnaia, Y. Bai, T. Bain, J. T. Baines, O. K. Baker, P. Balek, F. Balli, E. Banas, Sw. Banerjee, A. A. E. Bannoura, V. Bansal, H. S. Bansil, L. Barak, S. P. Baranov, E. L. Barberio, D. Barberis, M. Barbero, T. Barillari, M. Barisonzi, T. Barklow, N. Barlow, B. M. Barnett, R. M. Barnett, Z. Barnovska, A. Baroncelli, G. Barone, A. J. Barr, F. Barreiro, J. Barreiro Guimarães da Costa, R. Bartoldus, A. E. Barton, P. Bartos, V. Bartsch, A. Bassalat, A. Basye, R. L. Bates, J. R. Batley, M. Battaglia, M. Battistin, F. Bauer, H. S. Bawa, M. D. Beattie, T. Beau, P. H. Beauchemin, R. Beccherle, P. Bechtle, H. P. Beck, K. Becker, S. Becker, M. Beckingham, C. Becot, A. J. Beddall, A. Beddall, S. Bedikian, V. A. Bednyakov, C. P. Bee, L. J. Beemster, T. A. Beermann, M. Begel, J. K. Behr, C. Belanger-Champagne, P. J. Bell, W. H. Bell, G. Bella, L. Bellagamba, A. Bellerive, M. Bellomo, K. Belotskiy, O. Beltramello, O. Benary, D. Benchekroun, K. Bendtz, N. Benekos, Y. Benhammou, E. Benhar Noccioli, J. A. Benitez Garcia, D. P. Benjamin, J. R. Bensinger, K. Benslama, S. Bentvelsen, D. Berge, E. Bergeaas Kuutmann, N. Berger, F. Berghaus, J. Beringer, C. Bernard, P. Bernat, C. Bernius, F. U. Bernlochner, T. Berry, P. Berta, C. Bertella, G. Bertoli, F. Bertolucci, C. Bertsche, D. Bertsche, M. I. Besana, G. J. Besjes, O. Bessidskaia Bylund, M. Bessner, N. Besson, C. Betancourt, S. Bethke, W. Bhimji, R. M. Bianchi, L. Bianchini, M. Bianco, O. Biebel, S. P. Bieniek, K. Bierwagen, J. Biesiada, M. Biglietti, J. Bilbao De Mendizabal, H. Bilokon, M. Bindi, S. Binet, A. Bingul, C. Bini, C. W. Black, J. E. Black, K. M. Black, D. Blackburn, R. E. Blair, J.-B. Blanchard, T. Blazek, I. Bloch, C. Blocker, W. Blum, U. Blumenschein, G. J. Bobbink, V. S. Bobrovnikov, S. S. Bocchetta, A. Bocci, C. Bock, C. R. Boddy, M. Boehler, T. T. Boek, J. A. Bogaerts, A. G. Bogdanchikov, A. Bogouch, C. Bohm, J. Bohm, V. Boisvert, T. Bold, V. Boldea, A. S. Boldyrev, M. Bomben, M. Bona, M. Boonekamp, A. Borisov, G. Borissov, M. Borri, S. Borroni, J. Bortfeldt, V. Bortolotto, K. Bos, D. Boscherini, M. Bosman, H. Boterenbrood, J. Boudreau, J. Bouffard, E. V. Bouhova-Thacker, D. Boumediene, C. Bourdarios, N. Bousson, S. Boutouil, A. Boveia, J. Boyd, I. R. Boyko, I. Bozic, J. Bracinik, A. Brandt, G. Brandt, O. Brandt, U. Bratzler, B. Brau, J. E. Brau, H. M. Braun, S. F. Brazzale, B. Brelier, K. Brendlinger, A. J. Brennan, R. Brenner, S. Bressler, K. Bristow, T. M. Bristow, D. Britton, F. M. Brochu, I. Brock, R. Brock, C. Bromberg, J. Bronner, G. Brooijmans, T. Brooks, W. K. Brooks, J. Brosamer, E. Brost, J. Brown, P. A. Bruckman de Renstrom, D. Bruncko, R. Bruneliere, S. Brunet, A. Bruni, G. Bruni, M. Bruschi, L. Bryngemark, T. Buanes, Q. Buat, F. Bucci, P. Buchholz, R. M. Buckingham, A. G. Buckley, S. I. Buda, I. A. Budagov, F. Buehrer, L. Bugge, M. K. Bugge, O. Bulekov, A. C. Bundock, H. Burckhart, S. Burdin, B. Burghgrave, S. Burke, I. Burmeister, E. Busato, D. Büscher, V. Büscher, P. Bussey, C. P. Buszello, B. Butler, J. M. Butler, A. I. Butt, C. M. Buttar, J. M. Butterworth, P. Butti, W. Buttinger, A. Buzatu, M. Byszewski, S. Cabrera Urbán, D. Caforio, O. Cakir, P. Calafiura, A. Calandri, G. Calderini, P. Calfayan, R. Calkins, L. P. Caloba, D. Calvet, S. Calvet, R. Camacho Toro, S. Camarda, D. Cameron, L. M. Caminada, R. Caminal Armadans, S. Campana, M. Campanelli, A. Campoverde, V. Canale, A. Canepa, M. Cano Bret, J. Cantero, R. Cantrill, T. Cao, M. D. M. Capeans Garrido, I. Caprini, M. Caprini, M. Capua, R. Caputo, R. Cardarelli, T. Carli, G. Carlino, L. Carminati, S. Caron, E. Carquin, G. D. Carrillo-Montoya, J. R. Carter, J. Carvalho, D. Casadei, M. P. Casado, M. Casolino, E. Castaneda-Miranda, A. Castelli, V. Castillo Gimenez, N. F. Castro, P. Catastini, A. Catinaccio, J. R. Catmore, A. Cattai, G. Cattani, J. Caudron, V. Cavaliere, D. Cavalli, M. Cavalli-Sforza, V. Cavasinni, F. Ceradini, B. C. Cerio, K. Cerny, A. S. Cerqueira, A. Cerri, L. Cerrito, F. Cerutti, M. Cerv, A. Cervelli, S. A. Cetin, A. Chafaq, D. Chakraborty, I. Chalupkova, P. Chang, B. Chapleau, J. D. Chapman, D. Charfeddine, D. G. Charlton, C. C. Chau, C. A. Chavez Barajas, S. Cheatham, A. Chegwidden, S. Chekanov, S. V. Chekulaev, G. A. Chelkov, M. A. Chelstowska, C. Chen, H. Chen, K. Chen, L. Chen, S. Chen, X. Chen, Y. Chen, Y. Chen, H. C. Cheng, Y. Cheng, A. Cheplakov, R. Cherkaoui El Moursli, V. Chernyatin, E. Cheu, L. Chevalier, V. Chiarella, G. Chiefari, J. T. Childers, A. Chilingarov, G. Chiodini, A. S. Chisholm, R. T. Chislett, A. Chitan, M. V. Chizhov, S. Chouridou, B. K. B. Chow, D. Chromek-Burckhart, M. L. Chu, J. Chudoba, J. J. Chwastowski, L. Chytka, G. Ciapetti, A. K. Ciftci, R. Ciftci, D. Cinca, V. Cindro, A. Ciocio, P. Cirkovic, Z. H. Citron, M. Ciubancan, A. Clark, P. J. Clark, R. N. Clarke, W. Cleland, J. C. Clemens, C. Clement, Y. Coadou, M. Cobal, A. Coccaro, J. Cochran, L. Coffey, J. G. Cogan, J. Coggeshall, B. Cole, S. Cole, A. P. Colijn, J. Collot, T. Colombo, G. Colon, G. Compostella, P. Conde Muiño, E. Coniavitis, M. C. Conidi, S. H. Connell, I. A. Connelly, S. M. Consonni, V. Consorti, S. Constantinescu, C. Conta, G. Conti, F. Conventi, M. Cooke, B. D. Cooper, A. M. Cooper-Sarkar, N. J. Cooper-Smith, K. Copic, T. Cornelissen, M. Corradi, F. Corriveau, A. Corso-Radu, A. Cortes-Gonzalez, G. Cortiana, G. Costa, M. J. Costa, D. Costanzo, D. Côté, G. Cottin, G. Cowan, B. E. Cox, K. Cranmer, G. Cree, S. Crépé-Renaudin, F. Crescioli, W. A. Cribbs, M. Crispin Ortuzar, M. Cristinziani, V. Croft, G. Crosetti, C.-M. Cuciuc, T. Cuhadar Donszelmann, J. Cummings, M. Curatolo, C. Cuthbert, H. Czirr, P. Czodrowski, Z. Czyczula, S. D’Auria, M. D’Onofrio, M. J. Da Cunha Sargedas De Sousa, C. Da Via, W. Dabrowski, A. Dafinca, T. Dai, O. Dale, F. Dallaire, C. Dallapiccola, M. Dam, A. C. Daniells, M. Dano Hoffmann, V. Dao, G. Darbo, S. Darmora, J. Dassoulas, A. Dattagupta, W. Davey, C. David, T. Davidek, E. Davies, M. Davies, O. Davignon, A. R. Davison, P. Davison, Y. Davygora, E. Dawe, I. Dawson, R. K. Daya-Ishmukhametova, K. De, R. de Asmundis, S. De Castro, S. De Cecco, N. De Groot, P. de Jong, H. De la Torre, F. De Lorenzi, L. De Nooij, D. De Pedis, A. De Salvo, U. De Sanctis, A. De Santo, J. B. De Vivie De Regie, W. J. Dearnaley, R. Debbe, C. Debenedetti, B. Dechenaux, D. V. Dedovich, I. Deigaard, J. Del Peso, T. Del Prete, F. Deliot, C. M. Delitzsch, M. Deliyergiyev, A. Dell’Acqua, L. Dell’Asta, M. Dell’Orso, M. Della Pietra, D. della Volpe, M. Delmastro, P. A. Delsart, C. Deluca, S. Demers, M. Demichev, A. Demilly, S. P. Denisov, D. Derendarz, J. E. Derkaoui, F. Derue, P. Dervan, K. Desch, C. Deterre, P. O. Deviveiros, A. Dewhurst, S. Dhaliwal, A. Di Ciaccio, L. Di Ciaccio, A. Di Domenico, C. Di Donato, A. Di Girolamo, B. Di Girolamo, A. Di Mattia, B. Di Micco, R. Di Nardo, A. Di Simone, R. Di Sipio, D. Di Valentino, F. A. Dias, M. A. Diaz, E. B. Diehl, J. Dietrich, T. A. Dietzsch, S. Diglio, A. Dimitrievska, J. Dingfelder, C. Dionisi, P. Dita, S. Dita, F. Dittus, F. Djama, T. Djobava, J. I. Djuvsland, M. A. B. do Vale, A. Do Valle Wemans, D. Dobos, C. Doglioni, T. Doherty, T. Dohmae, J. Dolejsi, Z. Dolezal, B. A. Dolgoshein, M. Donadelli, S. Donati, P. Dondero, J. Donini, J. Dopke, A. Doria, M. T. Dova, A. T. Doyle, M. Dris, J. Dubbert, S. Dube, E. Dubreuil, E. Duchovni, G. Duckeck, O. A. Ducu, D. Duda, A. Dudarev, F. Dudziak, L. Duflot, L. Duguid, M. Dührssen, M. Dunford, H. Duran Yildiz, M. Düren, A. Durglishvili, M. Dwuznik, M. Dyndal, J. Ebke, W. Edson, N. C. Edwards, W. Ehrenfeld, T. Eifert, G. Eigen, K. Einsweiler, T. Ekelof, M. El Kacimi, M. Ellert, S. Elles, F. Ellinghaus, N. Ellis, J. Elmsheuser, M. Elsing, D. Emeliyanov, Y. Enari, O. C. Endner, M. Endo, R. Engelmann, J. Erdmann, A. Ereditato, D. Eriksson, G. Ernis, J. Ernst, M. Ernst, J. Ernwein, D. Errede, S. Errede, E. Ertel, M. Escalier, H. Esch, C. Escobar, B. Esposito, A. I. Etienvre, E. Etzion, H. Evans, A. Ezhilov, L. Fabbri, G. Facini, R. M. Fakhrutdinov, S. Falciano, R. J. Falla, J. Faltova, Y. Fang, M. Fanti, A. Farbin, A. Farilla, T. Farooque, S. Farrell, S. M. Farrington, P. Farthouat, F. Fassi, P. Fassnacht, D. Fassouliotis, A. Favareto, L. Fayard, P. Federic, O. L. Fedin, W. Fedorko, M. Fehling-Kaschek, S. Feigl, L. Feligioni, C. Feng, E. J. Feng, H. Feng, A. B. Fenyuk, S. Fernandez Perez, S. Ferrag, J. Ferrando, A. Ferrari, P. Ferrari, R. Ferrari, D. E. Ferreira de Lima, A. Ferrer, D. Ferrere, C. Ferretti, A. Ferretto Parodi, M. Fiascaris, F. Fiedler, A. Filipčič, M. Filipuzzi, F. Filthaut, M. Fincke-Keeler, K. D. Finelli, M. C. N. Fiolhais, L. Fiorini, A. Firan, A. Fischer, J. Fischer, W. C. Fisher, E. A. Fitzgerald, M. Flechl, I. Fleck, P. Fleischmann, S. Fleischmann, G. T. Fletcher, G. Fletcher, T. Flick, A. Floderus, L. R. Flores Castillo, A. C. Florez Bustos, M. J. Flowerdew, A. Formica, A. Forti, D. Fortin, D. Fournier, H. Fox, S. Fracchia, P. Francavilla, M. Franchini, S. Franchino, D. Francis, L. Franconi, M. Franklin, S. Franz, M. Fraternali, S. T. French, C. Friedrich, F. Friedrich, D. Froidevaux, J. A. Frost, C. Fukunaga, E. Fullana Torregrosa, B. G. Fulsom, J. Fuster, C. Gabaldon, O. Gabizon, A. Gabrielli, A. Gabrielli, S. Gadatsch, S. Gadomski, G. Gagliardi, P. Gagnon, C. Galea, B. Galhardo, E. J. Gallas, V. Gallo, B. J. Gallop, P. Gallus, G. Galster, K. K. Gan, J. Gao, Y. S. Gao, F. M. Garay Walls, F. Garberson, C. García, J. E. García Navarro, M. Garcia-Sciveres, R. W. Gardner, N. Garelli, V. Garonne, C. Gatti, G. Gaudio, B. Gaur, L. Gauthier, P. Gauzzi, I. L. Gavrilenko, C. Gay, G. Gaycken, E. N. Gazis, P. Ge, Z. Gecse, C. N. P. Gee, D. A. A. Geerts, Ch. Geich-Gimbel, K. Gellerstedt, C. Gemme, A. Gemmell, M. H. Genest, S. Gentile, M. George, S. George, D. Gerbaudo, A. Gershon, H. Ghazlane, N. Ghodbane, B. Giacobbe, S. Giagu, V. Giangiobbe, P. Giannetti, F. Gianotti, B. Gibbard, S. M. Gibson, M. Gilchriese, T. P. S. Gillam, D. Gillberg, G. Gilles, D. M. Gingrich, N. Giokaris, M. P. Giordani, R. Giordano, F. M. Giorgi, F. M. Giorgi, P. F. Giraud, D. Giugni, C. Giuliani, M. Giulini, B. K. Gjelsten, S. Gkaitatzis, I. Gkialas, L. K. Gladilin, C. Glasman, J. Glatzer, P. C. F. Glaysher, A. Glazov, G. L. Glonti, M. Goblirsch-Kolb, J. R. Goddard, J. Godlewski, C. Goeringer, S. Goldfarb, T. Golling, D. Golubkov, A. Gomes, L. S. Gomez Fajardo, R. Gonçalo, J. Goncalves Pinto Firmino Da Costa, L. Gonella, S. González de la Hoz, G. Gonzalez Parra, S. Gonzalez-Sevilla, L. Goossens, P. A. Gorbounov, H. A. Gordon, I. Gorelov, B. Gorini, E. Gorini, A. Gorišek, E. Gornicki, A. T. Goshaw, C. Gössling, M. I. Gostkin, M. Gouighri, D. Goujdami, M. P. Goulette, A. G. Goussiou, C. Goy, S. Gozpinar, H. M. X. Grabas, L. Graber, I. Grabowska-Bold, P. Grafström, K-J. Grahn, J. Gramling, E. Gramstad, S. Grancagnolo, V. Grassi, V. Gratchev, H. M. Gray, E. Graziani, O. G. Grebenyuk, Z. D. Greenwood, K. Gregersen, I. M. Gregor, P. Grenier, J. Griffiths, A. A. Grillo, K. Grimm, S. Grinstein, Ph. Gris, Y. V. Grishkevich, J.-F. Grivaz, J. P. Grohs, A. Grohsjean, E. Gross, J. Grosse-Knetter, G. C. Grossi, J. Groth-Jensen, Z. J. Grout, L. Guan, J. Guenther, F. Guescini, D. Guest, O. Gueta, C. Guicheney, E. Guido, T. Guillemin, S. Guindon, U. Gul, C. Gumpert, J. Guo, S. Gupta, P. Gutierrez, N. G. Gutierrez Ortiz, C. Gutschow, N. Guttman, C. Guyot, C. Gwenlan, C. B. Gwilliam, A. Haas, C. Haber, H. K. Hadavand, N. Haddad, P. Haefner, S. Hageböck, Z. Hajduk, H. Hakobyan, M. Haleem, D. Hall, G. Halladjian, K. Hamacher, P. Hamal, K. Hamano, M. Hamer, A. Hamilton, S. Hamilton, G. N. Hamity, P. G. Hamnett, L. Han, K. Hanagaki, K. Hanawa, M. Hance, P. Hanke, R. Hanna, J. B. Hansen, J. D. Hansen, P. H. Hansen, K. Hara, A. S. Hard, T. Harenberg, F. Hariri, S. Harkusha, D. Harper, R. D. Harrington, O. M. Harris, P. F. Harrison, F. Hartjes, M. Hasegawa, S. Hasegawa, Y. Hasegawa, A. Hasib, S. Hassani, S. Haug, M. Hauschild, R. Hauser, M. Havranek, C. M. Hawkes, R. J. Hawkings, A. D. Hawkins, T. Hayashi, D. Hayden, C. P. Hays, H. S. Hayward, S. J. Haywood, S. J. Head, T. Heck, V. Hedberg, L. Heelan, S. Heim, T. Heim, B. Heinemann, L. Heinrich, J. Hejbal, L. Helary, C. Heller, M. Heller, S. Hellman, D. Hellmich, C. Helsens, J. Henderson, R. C. W. Henderson, Y. Heng, C. Hengler, A. Henrichs, A. M. Henriques Correia, S. Henrot-Versille, C. Hensel, G. H. Herbert, Y. Hernández Jiménez, R. Herrberg-Schubert, G. Herten, R. Hertenberger, L. Hervas, G. G. Hesketh, N. P. Hessey, R. Hickling, E. Higón-Rodriguez, E. Hill, J. C. Hill, K. H. Hiller, S. Hillert, S. J. Hillier, I. Hinchliffe, E. Hines, M. Hirose, D. Hirschbuehl, J. Hobbs, N. Hod, M. C. Hodgkinson, P. Hodgson, A. Hoecker, M. R. Hoeferkamp, F. Hoenig, J. Hoffman, D. Hoffmann, M. Hohlfeld, T. R. Holmes, T. M. Hong, L. Hooft van Huysduynen, W. H. Hopkins, Y. Horii, J-Y. Hostachy, S. Hou, A. Hoummada, J. Howard, J. Howarth, M. Hrabovsky, I. Hristova, J. Hrivnac, T. Hryn’ova, C. Hsu, P. J. Hsu, S.-C. Hsu, D. Hu, X. Hu, Y. Huang, Z. Hubacek, F. Hubaut, F. Huegging, T. B. Huffman, E. W. Hughes, G. Hughes, M. Huhtinen, T. A. Hülsing, M. Hurwitz, N. Huseynov, J. Huston, J. Huth, G. Iacobucci, G. Iakovidis, I. Ibragimov, L. Iconomidou-Fayard, E. Ideal, P. Iengo, O. Igonkina, T. Iizawa, Y. Ikegami, K. Ikematsu, M. Ikeno, Y. Ilchenko, D. Iliadis, N. Ilic, Y. Inamaru, T. Ince, P. Ioannou, M. Iodice, K. Iordanidou, V. Ippolito, A. Irles Quiles, C. Isaksson, M. Ishino, M. Ishitsuka, R. Ishmukhametov, C. Issever, S. Istin, J. M. Iturbe Ponce, R. Iuppa, J. Ivarsson, W. Iwanski, H. Iwasaki, J. M. Izen, V. Izzo, B. Jackson, M. Jackson, P. Jackson, M. R. Jaekel, V. Jain, K. Jakobs, S. Jakobsen, T. Jakoubek, J. Jakubek, D. O. Jamin, D. K. Jana, E. Jansen, H. Jansen, J. Janssen, M. Janus, G. Jarlskog, N. Javadov, T. Javůrek, L. Jeanty, J. Jejelava, G.-Y. Jeng, D. Jennens, P. Jenni, J. Jentzsch, C. Jeske, S. Jézéquel, H. Ji, J. Jia, Y. Jiang, M. Jimenez Belenguer, S. Jin, A. Jinaru, O. Jinnouchi, M. D. Joergensen, K. E. Johansson, P. Johansson, K. A. Johns, K. Jon-And, G. Jones, R. W. L. Jones, T. J. Jones, J. Jongmanns, P. M. Jorge, K. D. Joshi, J. Jovicevic, X. Ju, C. A. Jung, R. M. Jungst, P. Jussel, A. Juste Rozas, M. Kaci, A. Kaczmarska, M. Kado, H. Kagan, M. Kagan, E. Kajomovitz, C. W. Kalderon, S. Kama, A. Kamenshchikov, N. Kanaya, M. Kaneda, S. Kaneti, V. A. Kantserov, J. Kanzaki, B. Kaplan, A. Kapliy, D. Kar, K. Karakostas, N. Karastathis, M. J. Kareem, M. Karnevskiy, S. N. Karpov, Z. M. Karpova, K. Karthik, V. Kartvelishvili, A. N. Karyukhin, L. Kashif, G. Kasieczka, R. D. Kass, A. Kastanas, Y. Kataoka, A. Katre, J. Katzy, V. Kaushik, K. Kawagoe, T. Kawamoto, G. Kawamura, S. Kazama, V. F. Kazanin, M. Y. Kazarinov, R. Keeler, R. Kehoe, J. S. Keller, J. J. Kempster, H. Keoshkerian, O. Kepka, B. P. Kerševan, S. Kersten, K. Kessoku, J. Keung, F. Khalil-zada, H. Khandanyan, A. Khanov, A. Khodinov, A. Khomich, T. J. Khoo, G. Khoriauli, A. Khoroshilov, V. Khovanskiy, E. Khramov, J. Khubua, H. Y. Kim, H. Kim, S. H. Kim, N. Kimura, O. M. Kind, B. T. King, M. King, R. S. B. King, S. B. King, J. Kirk, A. E. Kiryunin, T. Kishimoto, D. Kisielewska, F. Kiss, T. Kittelmann, K. Kiuchi, E. Kladiva, M. Klein, U. Klein, K. Kleinknecht, P. Klimek, A. Klimentov, R. Klingenberg, J. A. Klinger, T. Klioutchnikova, P. F. Klok, E.-E. Kluge, P. Kluit, S. Kluth, E. Kneringer, E. B. F. G. Knoops, A. Knue, D. Kobayashi, T. Kobayashi, M. Kobel, M. Kocian, P. Kodys, P. Koevesarki, T. Koffas, E. Koffeman, L. A. Kogan, S. Kohlmann, Z. Kohout, T. Kohriki, T. Koi, H. Kolanoski, I. Koletsou, J. Koll, A. A. Komar, Y. Komori, T. Kondo, N. Kondrashova, K. Köneke, A. C. König, S. König, T. Kono, R. Konoplich, N. Konstantinidis, R. Kopeliansky, S. Koperny, L. Köpke, A. K. Kopp, K. Korcyl, K. Kordas, A. Korn, A. A. Korol, I. Korolkov, E. V. Korolkova, V. A. Korotkov, O. Kortner, S. Kortner, V. V. Kostyukhin, V. M. Kotov, A. Kotwal, C. Kourkoumelis, V. Kouskoura, A. Koutsman, R. Kowalewski, T. Z. Kowalski, W. Kozanecki, A. S. Kozhin, V. Kral, V. A. Kramarenko, G. Kramberger, D. Krasnopevtsev, M. W. Krasny, A. Krasznahorkay, J. K. Kraus, A. Kravchenko, S. Kreiss, M. Kretz, J. Kretzschmar, K. Kreutzfeldt, P. Krieger, K. Kroeninger, H. Kroha, J. Kroll, J. Kroseberg, J. Krstic, U. Kruchonak, H. Krüger, T. Kruker, N. Krumnack, Z. V. Krumshteyn, A. Kruse, M. C. Kruse, M. Kruskal, T. Kubota, H. Kucuk, S. Kuday, S. Kuehn, A. Kugel, A. Kuhl, T. Kuhl, V. Kukhtin, Y. Kulchitsky, S. Kuleshov, M. Kuna, J. Kunkle, A. Kupco, H. Kurashige, Y. A. Kurochkin, R. Kurumida, V. Kus, E. S. Kuwertz, M. Kuze, J. Kvita, A. La Rosa, L. La Rotonda, C. Lacasta, F. Lacava, J. Lacey, H. Lacker, D. Lacour, V. R. Lacuesta, E. Ladygin, R. Lafaye, B. Laforge, T. Lagouri, S. Lai, H. Laier, L. Lambourne, S. Lammers, C. L. Lampen, W. Lampl, E. Lançon, U. Landgraf, M. P. J. Landon, V. S. Lang, A. J. Lankford, F. Lanni, K. Lantzsch, S. Laplace, C. Lapoire, J. F. Laporte, T. Lari, F. Lasagni Manghi, M. Lassnig, P. Laurelli, W. Lavrijsen, A. T. Law, P. Laycock, O. Le Dortz, E. Le Guirriec, E. Le Menedeu, T. LeCompte, F. Ledroit-Guillon, C. A. Lee, H. Lee, J. S. H. Lee, S. C. Lee, L. Lee, G. Lefebvre, M. Lefebvre, F. Legger, C. Leggett, A. Lehan, M. Lehmacher, G. Lehmann Miotto, X. Lei, W. A. Leight, A. Leisos, A. G. Leister, M. A. L. Leite, R. Leitner, D. Lellouch, B. Lemmer, K. J. C. Leney, T. Lenz, B. Lenzi, R. Leone, S. Leone, C. Leonidopoulos, S. Leontsinis, C. Leroy, C. G. Lester, C. M. Lester, M. Levchenko, J. Levêque, D. Levin, L. J. Levinson, M. Levy, A. Lewis, G. H. Lewis, A. M. Leyko, M. Leyton, B. Li, B. Li, H. Li, H. L. Li, L. Li, L. Li, S. Li, Y. Li, Z. Liang, H. Liao, B. Liberti, P. Lichard, K. Lie, J. Liebal, W. Liebig, C. Limbach, A. Limosani, S. C. Lin, T. H. Lin, F. Linde, B. E. Lindquist, J. T. Linnemann, E. Lipeles, A. Lipniacka, M. Lisovyi, T. M. Liss, D. Lissauer, A. Lister, A. M. Litke, B. Liu, D. Liu, J. B. Liu, K. Liu, L. Liu, M. Liu, M. Liu, Y. Liu, M. Livan, S. S. A. Livermore, A. Lleres, J. Llorente Merino, S. L. Lloyd, F. Lo Sterzo, E. Lobodzinska, P. Loch, W. S. Lockman, F. K. Loebinger, A. E. Loevschall-Jensen, A. Loginov, T. Lohse, K. Lohwasser, M. Lokajicek, V. P. Lombardo, B. A. Long, J. D. Long, R. E. Long, L. Lopes, D. Lopez Mateos, B. Lopez Paredes, I. Lopez Paz, J. Lorenz, N. Lorenzo Martinez, M. Losada, P. Loscutoff, X. Lou, A. Lounis, J. Love, P. A. Love, A. J. Lowe, N. Lu, H. J. Lubatti, C. Luci, A. Lucotte, F. Luehring, W. Lukas, L. Luminari, O. Lundberg, B. Lund-Jensen, M. Lungwitz, D. Lynn, R. Lysak, E. Lytken, H. Ma, L. L. Ma, G. Maccarrone, A. Macchiolo, J. Machado Miguens, D. Macina, D. Madaffari, R. Madar, H. J. Maddocks, W. F. Mader, A. Madsen, T. Maeno, M. Maeno Kataoka, A. Maevskiy, E. Magradze, K. Mahboubi, J. Mahlstedt, S. Mahmoud, C. Maiani, C. Maidantchik, A. A. Maier, A. Maio, S. Majewski, Y. Makida, N. Makovec, P. Mal, B. Malaescu, Pa. Malecki, V. P. Maleev, F. Malek, U. Mallik, D. Malon, C. Malone, S. Maltezos, V. M. Malyshev, S. Malyukov, J. Mamuzic, B. Mandelli, L. Mandelli, I. Mandić, R. Mandrysch, J. Maneira, A. Manfredini, L. Manhaes de Andrade Filho, J. Manjarres Ramos, A. Mann, P. M. Manning, A. Manousakis-Katsikakis, B. Mansoulie, R. Mantifel, L. Mapelli, L. March, J. F. Marchand, G. Marchiori, M. Marcisovsky, C. P. Marino, M. Marjanovic, C. N. Marques, F. Marroquim, S. P. Marsden, Z. Marshall, L. F. Marti, S. Marti-Garcia, B. Martin, B. Martin, T. A. Martin, V. J. Martin, B. Martin dit Latour, H. Martinez, M. Martinez, S. Martin-Haugh, A. C. Martyniuk, M. Marx, F. Marzano, A. Marzin, L. Masetti, T. Mashimo, R. Mashinistov, J. Masik, A. L. Maslennikov, I. Massa, L. Massa, N. Massol, P. Mastrandrea, A. Mastroberardino, T. Masubuchi, P. Mättig, J. Mattmann, J. Maurer, S. J. Maxfield, D. A. Maximov, R. Mazini, L. Mazzaferro, G. Mc Goldrick, S. P. Mc Kee, A. McCarn, R. L. McCarthy, T. G. McCarthy, N. A. McCubbin, K. W. McFarlane, J. A. Mcfayden, G. Mchedlidze, S. J. McMahon, R. A. McPherson, J. Mechnich, M. Medinnis, S. Meehan, S. Mehlhase, A. Mehta, K. Meier, C. Meineck, B. Meirose, C. Melachrinos, B. R. Mellado Garcia, F. Meloni, A. Mengarelli, S. Menke, E. Meoni, K. M. Mercurio, S. Mergelmeyer, N. Meric, P. Mermod, L. Merola, C. Meroni, F. S. Merritt, H. Merritt, A. Messina, J. Metcalfe, A. S. Mete, C. Meyer, C. Meyer, J-P. Meyer, J. Meyer, R. P. Middleton, S. Migas, L. Mijović, G. Mikenberg, M. Mikestikova, M. Mikuž, A. Milic, D. W. Miller, C. Mills, A. Milov, D. A. Milstead, D. Milstein, A. A. Minaenko, I. A. Minashvili, A. I. Mincer, B. Mindur, M. Mineev, Y. Ming, L. M. Mir, G. Mirabelli, T. Mitani, J. Mitrevski, V. A. Mitsou, S. Mitsui, A. Miucci, P. S. Miyagawa, J. U. Mjörnmark, T. Moa, K. Mochizuki, S. Mohapatra, W. Mohr, S. Molander, R. Moles-Valls, K. Mönig, C. Monini, J. Monk, E. Monnier, J. Montejo Berlingen, F. Monticelli, S. Monzani, R. W. Moore, N. Morange, D. Moreno, M. Moreno Llácer, P. Morettini, M. Morgenstern, M. Morii, S. Moritz, A. K. Morley, G. Mornacchi, J. D. Morris, L. Morvaj, H. G. Moser, M. Mosidze, J. Moss, K. Motohashi, R. Mount, E. Mountricha, S. V. Mouraviev, E. J. W. Moyse, S. Muanza, R. D. Mudd, F. Mueller, J. Mueller, K. Mueller, T. Mueller, T. Mueller, D. Muenstermann, Y. Munwes, J. A. Murillo Quijada, W. J. Murray, H. Musheghyan, E. Musto, A. G. Myagkov, M. Myska, O. Nackenhorst, J. Nadal, K. Nagai, R. Nagai, Y. Nagai, K. Nagano, A. Nagarkar, Y. Nagasaka, M. Nagel, A. M. Nairz, Y. Nakahama, K. Nakamura, T. Nakamura, I. Nakano, H. Namasivayam, G. Nanava, R. Narayan, T. Nattermann, T. Naumann, G. Navarro, R. Nayyar, H. A. Neal, P. Yu. Nechaeva, T. J. Neep, P. D. Nef, A. Negri, G. Negri, M. Negrini, S. Nektarijevic, C. Nellist, A. Nelson, T. K. Nelson, S. Nemecek, P. Nemethy, A. A. Nepomuceno, M. Nessi, M. S. Neubauer, M. Neumann, R. M. Neves, P. Nevski, P. R. Newman, D. H. Nguyen, R. B. Nickerson, R. Nicolaidou, B. Nicquevert, J. Nielsen, N. Nikiforou, A. Nikiforov, V. Nikolaenko, I. Nikolic-Audit, K. Nikolics, K. Nikolopoulos, P. Nilsson, Y. Ninomiya, A. Nisati, R. Nisius, T. Nobe, L. Nodulman, M. Nomachi, I. Nomidis, S. Norberg, M. Nordberg, O. Novgorodova, S. Nowak, M. Nozaki, L. Nozka, K. Ntekas, G. Nunes Hanninger, T. Nunnemann, E. Nurse, F. Nuti, B. J. O’Brien, F. O’grady, D. C. O’Neil, V. O’Shea, F. G. Oakham, H. Oberlack, T. Obermann, J. Ocariz, A. Ochi, I. Ochoa, S. Oda, S. Odaka, H. Ogren, A. Oh, S. H. Oh, C. C. Ohm, H. Ohman, W. Okamura, H. Okawa, Y. Okumura, T. Okuyama, A. Olariu, A. G. Olchevski, S. A. Olivares Pino, D. Oliveira Damazio, E. Oliver Garcia, A. Olszewski, J. Olszowska, A. Onofre, P. U. E. Onyisi, C. J. Oram, M. J. Oreglia, Y. Oren, D. Orestano, N. Orlando, C. Oropeza Barrera, R. S. Orr, B. Osculati, R. Ospanov, G. Otero y Garzon, H. Otono, M. Ouchrif, E. A. Ouellette, F. Ould-Saada, A. Ouraou, K. P. Oussoren, Q. Ouyang, A. Ovcharova, M. Owen, V. E. Ozcan, N. Ozturk, K. Pachal, A. Pacheco Pages, C. Padilla Aranda, M. Pagáčová, S. Pagan Griso, E. Paganis, C. Pahl, F. Paige, P. Pais, K. Pajchel, G. Palacino, S. Palestini, M. Palka, D. Pallin, A. Palma, J. D. Palmer, Y. B. Pan, E. Panagiotopoulou, J. G. Panduro Vazquez, P. Pani, N. Panikashvili, S. Panitkin, D. Pantea, L. Paolozzi, Th. D. Papadopoulou, K. Papageorgiou, A. Paramonov, D. Paredes Hernandez, M. A. Parker, F. Parodi, J. A. Parsons, U. Parzefall, E. Pasqualucci, S. Passaggio, A. Passeri, F. Pastore, Fr. Pastore, G. Pásztor, S. Pataraia, N. D. Patel, J. R. Pater, S. Patricelli, T. Pauly, J. Pearce, L. E. Pedersen, M. Pedersen, S. Pedraza Lopez, R. Pedro, S. V. Peleganchuk, D. Pelikan, H. Peng, B. Penning, J. Penwell, D. V. Perepelitsa, E. Perez Codina, M. T. Pérez García-Estañ, V. Perez Reale, L. Perini, H. Pernegger, S. Perrella, R. Perrino, R. Peschke, V. D. Peshekhonov, K. Peters, R. F. Y. Peters, B. A. Petersen, T. C. Petersen, E. Petit, A. Petridis, C. Petridou, E. Petrolo, F. Petrucci, N. E. Pettersson, R. Pezoa, P. W. Phillips, G. Piacquadio, E. Pianori, A. Picazio, E. Piccaro, M. Piccinini, R. Piegaia, D. T. Pignotti, J. E. Pilcher, A. D. Pilkington, J. Pina, M. Pinamonti, A. Pinder, J. L. Pinfold, A. Pingel, B. Pinto, S. Pires, M. Pitt, C. Pizio, L. Plazak, M.-A. Pleier, V. Pleskot, E. Plotnikova, P. Plucinski, S. Poddar, F. Podlyski, R. Poettgen, L. Poggioli, D. Pohl, M. Pohl, G. Polesello, A. Policicchio, R. Polifka, A. Polini, C. S. Pollard, V. Polychronakos, K. Pommès, L. Pontecorvo, B. G. Pope, G. A. Popeneciu, D. S. Popovic, A. Poppleton, X. Portell Bueso, S. Pospisil, K. Potamianos, I. N. Potrap, C. J. Potter, C. T. Potter, G. Poulard, J. Poveda, V. Pozdnyakov, P. Pralavorio, A. Pranko, S. Prasad, R. Pravahan, S. Prell, D. Price, J. Price, L. E. Price, D. Prieur, M. Primavera, M. Proissl, K. Prokofiev, F. Prokoshin, E. Protopapadaki, S. Protopopescu, J. Proudfoot, M. Przybycien, H. Przysiezniak, E. Ptacek, D. Puddu, E. Pueschel, D. Puldon, M. Purohit, P. Puzo, J. Qian, G. Qin, Y. Qin, A. Quadt, D. R. Quarrie, W. B. Quayle, M. Queitsch-Maitland, D. Quilty, A. Qureshi, V. Radeka, V. Radescu, S. K. Radhakrishnan, P. Radloff, P. Rados, F. Ragusa, G. Rahal, S. Rajagopalan, M. Rammensee, A. S. Randle-Conde, C. Rangel-Smith, K. Rao, F. Rauscher, T. C. Rave, T. Ravenscroft, M. Raymond, A. L. Read, N. P. Readioff, D. M. Rebuzzi, A. Redelbach, G. Redlinger, R. Reece, K. Reeves, L. Rehnisch, H. Reisin, M. Relich, C. Rembser, H. Ren, Z. L. Ren, A. Renaud, M. Rescigno, S. Resconi, O. L. Rezanova, P. Reznicek, R. Rezvani, R. Richter, M. Ridel, P. Rieck, J. Rieger, M. Rijssenbeek, A. Rimoldi, L. Rinaldi, E. Ritsch, I. Riu, F. Rizatdinova, E. Rizvi, S. H. Robertson, A. Robichaud-Veronneau, D. Robinson, J. E. M. Robinson, A. Robson, C. Roda, L. Rodrigues, S. Roe, O. Røhne, S. Rolli, A. Romaniouk, M. Romano, E. Romero Adam, N. Rompotis, M. Ronzani, L. Roos, E. Ros, S. Rosati, K. Rosbach, M. Rose, P. Rose, P. L. Rosendahl, O. Rosenthal, V. Rossetti, E. Rossi, L. P. Rossi, R. Rosten, M. Rotaru, I. Roth, J. Rothberg, D. Rousseau, C. R. Royon, A. Rozanov, Y. Rozen, X. Ruan, F. Rubbo, I. Rubinskiy, V. I. Rud, C. Rudolph, M. S. Rudolph, F. Rühr, A. Ruiz-Martinez, Z. Rurikova, N. A. Rusakovich, A. Ruschke, J. P. Rutherfoord, N. Ruthmann, Y. F. Ryabov, M. Rybar, G. Rybkin, N. C. Ryder, A. F. Saavedra, S. Sacerdoti, A. Saddique, I. Sadeh, H. F-W. Sadrozinski, R. Sadykov, F. Safai Tehrani, H. Sakamoto, Y. Sakurai, G. Salamanna, A. Salamon, M. Saleem, D. Salek, P. H. Sales De Bruin, D. Salihagic, A. Salnikov, J. Salt, D. Salvatore, F. Salvatore, A. Salvucci, A. Salzburger, D. Sampsonidis, A. Sanchez, J. Sánchez, V. Sanchez Martinez, H. Sandaker, R. L. Sandbach, H. G. Sander, M. P. Sanders, M. Sandhoff, T. Sandoval, C. Sandoval, R. Sandstroem, D. P. C. Sankey, A. Sansoni, C. Santoni, R. Santonico, H. Santos, I. Santoyo Castillo, K. Sapp, A. Sapronov, J. G. Saraiva, E. Sarkisyan-Grinbaum, B. Sarrazin, G. Sartisohn, O. Sasaki, Y. Sasaki, G. Sauvage, E. Sauvan, P. Savard, D. O. Savu, C. Sawyer, L. Sawyer, D. H. Saxon, J. Saxon, C. Sbarra, A. Sbrizzi, T. Scanlon, D. A. Scannicchio, M. Scarcella, V. Scarfone, J. Schaarschmidt, P. Schacht, D. Schaefer, R. Schaefer, S. Schaepe, S. Schaetzel, U. Schäfer, A. C. Schaffer, D. Schaile, R. D. Schamberger, V. Scharf, V. A. Schegelsky, D. Scheirich, M. Schernau, M. I. Scherzer, C. Schiavi, J. Schieck, C. Schillo, M. Schioppa, S. Schlenker, E. Schmidt, K. Schmieden, C. Schmitt, S. Schmitt, B. Schneider, Y. J. Schnellbach, U. Schnoor, L. Schoeffel, A. Schoening, B. D. Schoenrock, A. L. S. Schorlemmer, M. Schott, D. Schouten, J. Schovancova, S. Schramm, M. Schreyer, C. Schroeder, N. Schuh, M. J. Schultens, H.-C. Schultz-Coulon, H. Schulz, M. Schumacher, B. A. Schumm, Ph. Schune, C. Schwanenberger, A. Schwartzman, T. A. Schwarz, Ph. Schwegler, Ph. Schwemling, R. Schwienhorst, J. Schwindling, T. Schwindt, M. Schwoerer, F. G. Sciacca, E. Scifo, G. Sciolla, W. G. Scott, F. Scuri, F. Scutti, J. Searcy, G. Sedov, E. Sedykh, S. C. Seidel, A. Seiden, F. Seifert, J. M. Seixas, G. Sekhniaidze, S. J. Sekula, K. E. Selbach, D. M. Seliverstov, G. Sellers, N. Semprini-Cesari, C. Serfon, L. Serin, L. Serkin, T. Serre, R. Seuster, H. Severini, T. Sfiligoj, F. Sforza, A. Sfyrla, E. Shabalina, M. Shamim, L. Y. Shan, R. Shang, J. T. Shank, M. Shapiro, P. B. Shatalov, K. Shaw, C. Y. Shehu, P. Sherwood, L. Shi, S. Shimizu, C. O. Shimmin, M. Shimojima, M. Shiyakova, A. Shmeleva, M. J. Shochet, D. Short, S. Shrestha, E. Shulga, M. A. Shupe, S. Shushkevich, P. Sicho, O. Sidiropoulou, D. Sidorov, A. Sidoti, F. Siegert, Dj. Sijacki, J. Silva, Y. Silver, D. Silverstein, S. B. Silverstein, V. Simak, O. Simard, Lj. Simic, S. Simion, E. Simioni, B. Simmons, R. Simoniello, M. Simonyan, P. Sinervo, N. B. Sinev, V. Sipica, G. Siragusa, A. Sircar, A. N. Sisakyan, S. Yu. Sivoklokov, J. Sjölin, T. B. Sjursen, H. P. Skottowe, K. Yu. Skovpen, P. Skubic, M. Slater, T. Slavicek, K. Sliwa, V. Smakhtin, B. H. Smart, L. Smestad, S. Yu. Smirnov, Y. Smirnov, L. N. Smirnova, O. Smirnova, K. M. Smith, M. Smizanska, K. Smolek, A. A. Snesarev, G. Snidero, S. Snyder, R. Sobie, F. Socher, A. Soffer, D. A. Soh, C. A. Solans, M. Solar, J. Solc, E. Yu. Soldatov, U. Soldevila, A. A. Solodkov, A. Soloshenko, O. V. Solovyanov, V. Solovyev, P. Sommer, H. Y. Song, N. Soni, A. Sood, A. Sopczak, B. Sopko, V. Sopko, V. Sorin, M. Sosebee, R. Soualah, P. Soueid, A. M. Soukharev, D. South, S. Spagnolo, F. Spanò, W. R. Spearman, F. Spettel, R. Spighi, G. Spigo, L. A. Spiller, M. Spousta, T. Spreitzer, B. Spurlock, R. D. St. Denis, S. Staerz, J. Stahlman, R. Stamen, S. Stamm, E. Stanecka, R. W. Stanek, C. Stanescu, M. Stanescu-Bellu, M. M. Stanitzki, S. Stapnes, E. A. Starchenko, J. Stark, P. Staroba, P. Starovoitov, R. Staszewski, P. Stavina, P. Steinberg, B. Stelzer, H. J. Stelzer, O. Stelzer-Chilton, H. Stenzel, S. Stern, G. A. Stewart, J. A. Stillings, M. C. Stockton, M. Stoebe, G. Stoicea, P. Stolte, S. Stonjek, A. R. Stradling, A. Straessner, M. E. Stramaglia, J. Strandberg, S. Strandberg, A. Strandlie, E. Strauss, M. Strauss, P. Strizenec, R. Ströhmer, D. M. Strom, R. Stroynowski, A. Strubig, S. A. Stucci, B. Stugu, N. A. Styles, D. Su, J. Su, R. Subramaniam, A. Succurro, Y. Sugaya, C. Suhr, M. Suk, V. V. Sulin, S. Sultansoy, T. Sumida, S. Sun, X. Sun, J. E. Sundermann, K. Suruliz, G. Susinno, M. R. Sutton, Y. Suzuki, M. Svatos, S. Swedish, M. Swiatlowski, I. Sykora, T. Sykora, D. Ta, C. Taccini, K. Tackmann, J. Taenzer, A. Taffard, R. Tafirout, N. Taiblum, H. Takai, R. Takashima, H. Takeda, T. Takeshita, Y. Takubo, M. Talby, A. A. Talyshev, J. Y. C. Tam, K. G. Tan, J. Tanaka, R. Tanaka, S. Tanaka, S. Tanaka, A. J. Tanasijczuk, B. B. Tannenwald, N. Tannoury, S. Tapprogge, S. Tarem, F. Tarrade, G. F. Tartarelli, P. Tas, M. Tasevsky, T. Tashiro, E. Tassi, A. Tavares Delgado, Y. Tayalati, F. E. Taylor, G. N. Taylor, W. Taylor, F. A. Teischinger, M. Teixeira Dias Castanheira, P. Teixeira-Dias, K. K. Temming, H. Ten Kate, P. K. Teng, J. J. Teoh, S. Terada, K. Terashi, J. Terron, S. Terzo, M. Testa, R. J. Teuscher, J. Therhaag, T. Theveneaux-Pelzer, J. P. Thomas, J. Thomas-Wilsker, E. N. Thompson, P. D. Thompson, P. D. Thompson, R. J. Thompson, A. S. Thompson, L. A. Thomsen, E. Thomson, M. Thomson, W. M. Thong, R. P. Thun, F. Tian, M. J. Tibbetts, V. O. Tikhomirov, Yu. A. Tikhonov, S. Timoshenko, E. Tiouchichine, P. Tipton, S. Tisserant, T. Todorov, S. Todorova-Nova, B. Toggerson, J. Tojo, S. Tokár, K. Tokushuku, K. Tollefson, E. Tolley, L. Tomlinson, M. Tomoto, L. Tompkins, K. Toms, N. D. Topilin, E. Torrence, H. Torres, E. Torró Pastor, J. Toth, F. Touchard, D. R. Tovey, H. L. Tran, T. Trefzger, L. Tremblet, A. Tricoli, I. M. Trigger, S. Trincaz-Duvoid, M. F. Tripiana, W. Trischuk, B. Trocmé, C. Troncon, M. Trottier-McDonald, M. Trovatelli, P. True, M. Trzebinski, A. Trzupek, C. Tsarouchas, J. C-L. Tseng, P. V. Tsiareshka, D. Tsionou, G. Tsipolitis, N. Tsirintanis, S. Tsiskaridze, V. Tsiskaridze, E. G. Tskhadadze, I. I. Tsukerman, V. Tsulaia, S. Tsuno, D. Tsybychev, A. Tudorache, V. Tudorache, A. N. Tuna, S. A. Tupputi, S. Turchikhin, D. Turecek, R. Turra, P. M. Tuts, A. Tykhonov, M. Tylmad, M. Tyndel, K. Uchida, I. Ueda, R. Ueno, M. Ughetto, M. Ugland, M. Uhlenbrock, F. Ukegawa, G. Unal, A. Undrus, G. Unel, F. C. Ungaro, Y. Unno, C. Unverdorben, D. Urbaniec, P. Urquijo, G. Usai, A. Usanova, L. Vacavant, V. Vacek, B. Vachon, N. Valencic, S. Valentinetti, A. Valero, L. Valery, S. Valkar, E. Valladolid Gallego, S. Vallecorsa, J. A. Valls Ferrer, W. Van Den Wollenberg, P. C. Van Der Deijl, R. van der Geer, H. van der Graaf, R. Van Der Leeuw, D. van der Ster, N. van Eldik, P. van Gemmeren, J. Van Nieuwkoop, I. van Vulpen, M. C. van Woerden, M. Vanadia, W. Vandelli, R. Vanguri, A. Vaniachine, F. Vannucci, G. Vardanyan, R. Vari, E. W. Varnes, T. Varol, D. Varouchas, A. Vartapetian, K. E. Varvell, F. Vazeille, T. Vazquez Schroeder, J. Veatch, F. Veloso, T. Velz, S. Veneziano, A. Ventura, D. Ventura, M. Venturi, N. Venturi, A. Venturini, V. Vercesi, M. Verducci, W. Verkerke, J. C. Vermeulen, A. Vest, M. C. Vetterli, O. Viazlo, I. Vichou, T. Vickey, O. E. Vickey Boeriu, G. H. A. Viehhauser, S. Viel, R. Vigne, M. Villa, M. Villaplana Perez, E. Vilucchi, M. G. Vincter, V. B. Vinogradov, J. Virzi, I. Vivarelli, F. Vives Vaque, S. Vlachos, D. Vladoiu, M. Vlasak, A. Vogel, M. Vogel, P. Vokac, G. Volpi, M. Volpi, H. von der Schmitt, H. von Radziewski, E. von Toerne, V. Vorobel, K. Vorobev, M. Vos, R. Voss, J. H. Vossebeld, N. Vranjes, M. Vranjes Milosavljevic, V. Vrba, M. Vreeswijk, T. Vu Anh, R. Vuillermet, I. Vukotic, Z. Vykydal, P. Wagner, W. Wagner, H. Wahlberg, S. Wahrmund, J. Wakabayashi, J. Walder, R. Walker, W. Walkowiak, R. Wall, P. Waller, B. Walsh, C. Wang, C. Wang, F. Wang, H. Wang, H. Wang, J. Wang, J. Wang, K. Wang, R. Wang, S. M. Wang, T. Wang, X. Wang, C. Wanotayaroj, A. Warburton, C. P. Ward, D. R. Wardrope, M. Warsinsky, A. Washbrook, C. Wasicki, P. M. Watkins, A. T. Watson, I. J. Watson, M. F. Watson, G. Watts, S. Watts, B. M. Waugh, S. Webb, M. S. Weber, S. W. Weber, J. S. Webster, A. R. Weidberg, P. Weigell, B. Weinert, J. Weingarten, C. Weiser, H. Weits, P. S. Wells, T. Wenaus, D. Wendland, Z. Weng, T. Wengler, S. Wenig, N. Wermes, M. Werner, P. Werner, M. Wessels, J. Wetter, K. Whalen, A. White, M. J. White, R. White, S. White, D. Whiteson, D. Wicke, F. J. Wickens, W. Wiedenmann, M. Wielers, P. Wienemann, C. Wiglesworth, L. A. M. Wiik-Fuchs, P. A. Wijeratne, A. Wildauer, M. A. Wildt, H. G. Wilkens, J. Z. Will, H. H. Williams, S. Williams, C. Willis, S. Willocq, A. Wilson, J. A. Wilson, I. Wingerter-Seez, F. Winklmeier, B. T. Winter, M. Wittgen, T. Wittig, J. Wittkowski, S. J. Wollstadt, M. W. Wolter, H. Wolters, B. K. Wosiek, J. Wotschack, M. J. Woudstra, K. W. Wozniak, M. Wright, M. Wu, S. L. Wu, X. Wu, Y. Wu, E. Wulf, T. R. Wyatt, B. M. Wynne, S. Xella, M. Xiao, D. Xu, L. Xu, B. Yabsley, S. Yacoob, R. Yakabe, M. Yamada, H. Yamaguchi, Y. Yamaguchi, A. Yamamoto, K. Yamamoto, S. Yamamoto, T. Yamamura, T. Yamanaka, K. Yamauchi, Y. Yamazaki, Z. Yan, H. Yang, H. Yang, U. K. Yang, Y. Yang, S. Yanush, L. Yao, W-M. Yao, Y. Yasu, E. Yatsenko, K. H. Yau Wong, J. Ye, S. Ye, I. Yeletskikh, A. L. Yen, E. Yildirim, M. Yilmaz, R. Yoosoofmiya, K. Yorita, R. Yoshida, K. Yoshihara, C. Young, C. J. S. Young, S. Youssef, D. R. Yu, J. Yu, J. M. Yu, J. Yu, L. Yuan, A. Yurkewicz, I. Yusuff, B. Zabinski, R. Zaidan, A. M. Zaitsev, A. Zaman, S. Zambito, L. Zanello, D. Zanzi, C. Zeitnitz, M. Zeman, A. Zemla, K. Zengel, O. Zenin, T. Ženiš, D. Zerwas, G. Zevi della Porta, D. Zhang, F. Zhang, H. Zhang, J. Zhang, L. Zhang, X. Zhang, Z. Zhang, Z. Zhao, A. Zhemchugov, J. Zhong, B. Zhou, L. Zhou, N. Zhou, C. G. Zhu, H. Zhu, J. Zhu, Y. Zhu, X. Zhuang, K. Zhukov, A. Zibell, D. Zieminska, N. I. Zimine, C. Zimmermann, R. Zimmermann, S. Zimmermann, S. Zimmermann, Z. Zinonos, M. Ziolkowski, G. Zobernig, A. Zoccoli, M. zur Nedden, G. Zurzolo, V. Zutshi, L. Zwalinski, ATLAS Collaboration

**Affiliations:** Department of Physics, University of Adelaide, Adelaide, Australia; Physics Department, SUNY Albany, Albany, NY USA; Department of Physics, University of Alberta, Edmonton, AB Canada; Department of Physics, Ankara University, Ankara, Turkey; Department of Physics, Gazi University, Ankara, Turkey; Division of Physics, TOBB University of Economics and Technology, Ankara, Turkey; Turkish Atomic Energy Authority, Ankara, Turkey; LAPP, CNRS/IN2P3 and Université Savoie Mont Blanc, Annecy-le-Vieux, France; High Energy Physics Division, Argonne National Laboratory, Argonne, IL USA; Department of Physics, University of Arizona, Tucson, AZ USA; Department of Physics, The University of Texas at Arlington, Arlington, TX USA; Physics Department, University of Athens, Athens, Greece; Physics Department, National Technical University of Athens, Zografou, Greece; Institute of Physics, Azerbaijan Academy of Sciences, Baku, Azerbaijan; Institut de Física d’Altes Energies and Departament de Física de la Universitat Autònoma de Barcelona, Barcelona, Spain; Institute of Physics, University of Belgrade, Belgrade, Serbia; Department for Physics and Technology, University of Bergen, Bergen, Norway; Physics Division, Lawrence Berkeley National Laboratory and University of California, Berkeley, CA USA; Department of Physics, Humboldt University, Berlin, Germany; Albert Einstein Center for Fundamental Physics and Laboratory for High Energy Physics, University of Bern, Bern, Switzerland; School of Physics and Astronomy, University of Birmingham, Birmingham, UK; Department of Physics, Bogazici University, Istanbul, Turkey; Department of Physics, Dogus University, Istanbul, Turkey; Department of Physics Engineering, Gaziantep University, Gaziantep, Turkey; INFN Sezione di Bologna, Bologna, Italy; Dipartimento di Fisica e Astronomia, Università di Bologna, Bologna, Italy; Physikalisches Institut, University of Bonn, Bonn, Germany; Department of Physics, Boston University, Boston, MA USA; Department of Physics, Brandeis University, Waltham, MA USA; Universidade Federal do Rio De Janeiro COPPE/EE/IF, Rio de Janeiro, Brazil; Electrical Circuits Department, Federal University of Juiz de Fora (UFJF), Juiz de Fora, Brazil; Federal University of Sao Joao del Rei (UFSJ), Sao Joao del Rei, Brazil; Instituto de Fisica, Universidade de Sao Paulo, São Paulo, Brazil; Physics Department, Brookhaven National Laboratory, Upton, NY USA; National Institute of Physics and Nuclear Engineering, Bucharest, Romania; Physics Department, National Institute for Research and Development of Isotopic and Molecular Technologies, Cluj Napoca, Romania; University Politehnica Bucharest, Bucharest, Romania; West University in Timisoara, Timisoara, Romania; Departamento de Física, Universidad de Buenos Aires, Buenos Aires, Argentina; Cavendish Laboratory, University of Cambridge, Cambridge, UK; Department of Physics, Carleton University, Ottawa, ON Canada; CERN, Geneva, Switzerland; Enrico Fermi Institute, University of Chicago, Chicago, IL USA; Departamento de Física, Pontificia Universidad Católica de Chile, Santiago, Chile; Departamento de Física, Universidad Técnica Federico Santa María, Valparaiso, Chile; Institute of High Energy Physics, Chinese Academy of Sciences, Beijing, China; Department of Modern Physics, University of Science and Technology of China, Hefei, Anhui China; Department of Physics, Nanjing University, Nanjing, Jiangsu China; School of Physics, Shandong University, Shandong, China; Shanghai Key Laboratory for Particle Physics and Cosmology, Department of Physics and Astronomy, Shanghai Jiao Tong University, Shanghai, China; Laboratoire de Physique Corpusculaire, Clermont Université and Université Blaise Pascal and CNRS/IN2P3, Clermont-Ferrand, France; Nevis Laboratory, Columbia University, Irvington, NY USA; Niels Bohr Institute, University of Copenhagen, Copenhagen, Denmark; INFN Gruppo Collegato di Cosenza, Laboratori Nazionali di Frascati, Frascati, Italy; Dipartimento di Fisica, Università della Calabria, Rende, Italy; AGH University of Science and Technology, Faculty of Physics and Applied Computer Science, Kraków, Poland; Marian Smoluchowski Institute of Physics, Jagiellonian University, Kraków, Poland; Institute of Nuclear Physics, Polish Academy of Sciences, Kraków, Poland; Physics Department, Southern Methodist University, Dallas, TX USA; Physics Department, University of Texas at Dallas, Richardson, TX USA; DESY, Hamburg and Zeuthen, Germany; Institut für Experimentelle Physik IV, Technische Universität Dortmund, Dortmund, Germany; Institut für Kern- und Teilchenphysik, Technische Universität Dresden, Dresden, Germany; Department of Physics, Duke University, Durham, NC USA; SUPA-School of Physics and Astronomy, University of Edinburgh, Edinburgh, UK; INFN Laboratori Nazionali di Frascati, Frascati, Italy; Fakultät für Mathematik und Physik, Albert-Ludwigs-Universität, Freiburg, Germany; Section de Physique, Université de Genève, Geneva, Switzerland; INFN Sezione di Genova, Genoa, Italy; Dipartimento di Fisica, Università di Genova, Genoa, Italy; E. Andronikashvili Institute of Physics, Iv. Javakhishvili Tbilisi State University, Tbilisi, Georgia; High Energy Physics Institute, Tbilisi State University, Tbilisi, Georgia; II Physikalisches Institut, Justus-Liebig-Universität Giessen, Giessen, Germany; SUPA-School of Physics and Astronomy, University of Glasgow, Glasgow, UK; II Physikalisches Institut, Georg-August-Universität, Göttingen, Germany; Laboratoire de Physique Subatomique et de Cosmologie, Université Grenoble-Alpes, CNRS/IN2P3, Grenoble, France; Department of Physics, Hampton University, Hampton, VA USA; Laboratory for Particle Physics and Cosmology, Harvard University, Cambridge, MA USA; Kirchhoff-Institut für Physik, Ruprecht-Karls-Universität Heidelberg, Heidelberg, Germany; Physikalisches Institut, Ruprecht-Karls-Universität Heidelberg, Heidelberg, Germany; ZITI Institut für technische Informatik, Ruprecht-Karls-Universität Heidelberg, Mannheim, Germany; Faculty of Applied Information Science, Hiroshima Institute of Technology, Hiroshima, Japan; Department of Physics, Indiana University, Bloomington, IN USA; Institut für Astro- und Teilchenphysik, Leopold-Franzens-Universität, Innsbruck, Austria; University of Iowa, Iowa City, IA USA; Department of Physics and Astronomy, Iowa State University, Ames, IA USA; Joint Institute for Nuclear Research, JINR Dubna, Dubna, Russia; KEK, High Energy Accelerator Research Organization, Tsukuba, Japan; Graduate School of Science, Kobe University, Kobe, Japan; Faculty of Science, Kyoto University, Kyoto, Japan; Kyoto University of Education, Kyoto, Japan; Department of Physics, Kyushu University, Fukuoka, Japan; Instituto de Física La Plata, Universidad Nacional de La Plata and CONICET, La Plata, Argentina; Physics Department, Lancaster University, Lancaster, UK; INFN Sezione di Lecce, Lecce, Italy; Dipartimento di Matematica e Fisica, Università del Salento, Lecce, Italy; Oliver Lodge Laboratory, University of Liverpool, Liverpool, UK; Department of Physics, Jožef Stefan Institute and University of Ljubljana, Ljubljana, Slovenia; School of Physics and Astronomy, Queen Mary University of London, London, UK; Department of Physics, Royal Holloway University of London, Surrey, UK; Department of Physics and Astronomy, University College London, London, UK; Louisiana Tech University, Ruston, LA USA; Laboratoire de Physique Nucléaire et de Hautes Energies, UPMC and Université Paris-Diderot and CNRS/IN2P3, Paris, France; Fysiska institutionen, Lunds universitet, Lund, Sweden; Departamento de Fisica Teorica C-15, Universidad Autonoma de Madrid, Madrid, Spain; Institut für Physik, Universität Mainz, Mainz, Germany; School of Physics and Astronomy, University of Manchester, Manchester, UK; CPPM, Aix-Marseille Université and CNRS/IN2P3, Marseille, France; Department of Physics, University of Massachusetts, Amherst, MA USA; Department of Physics, McGill University, Montreal, QC Canada; School of Physics, University of Melbourne, Melbourne, VIC Australia; Department of Physics, The University of Michigan, Ann Arbor, MI USA; Department of Physics and Astronomy, Michigan State University, East Lansing, MI USA; INFN Sezione di Milano, Milan, Italy; Dipartimento di Fisica, Università di Milano, Milan, Italy; B.I. Stepanov Institute of Physics, National Academy of Sciences of Belarus, Minsk, Republic of Belarus; National Scientific and Educational Centre for Particle and High Energy Physics, Minsk, Republic of Belarus; Department of Physics, Massachusetts Institute of Technology, Cambridge, MA USA; Group of Particle Physics, University of Montreal, Montreal, QC Canada; P.N. Lebedev Institute of Physics, Academy of Sciences, Moscow, Russia; Institute for Theoretical and Experimental Physics (ITEP), Moscow, Russia; National Research Nuclear University MEPhI, Moscow, Russia; D.V. Skobeltsyn Institute of Nuclear Physics, M.V. Lomonosov Moscow State University, Moscow, Russia; Fakultät für Physik, Ludwig-Maximilians-Universität München, Munich, Germany; Max-Planck-Institut für Physik (Werner-Heisenberg-Institut), Munich, Germany; Nagasaki Institute of Applied Science, Nagasaki, Japan; Graduate School of Science and Kobayashi-Maskawa Institute, Nagoya University, Nagoya, Japan; INFN Sezione di Napoli, Naples, Italy; Dipartimento di Fisica, Università di Napoli, Naples, Italy; Department of Physics and Astronomy, University of New Mexico, Albuquerque, NM USA; Institute for Mathematics, Astrophysics and Particle Physics, Radboud University Nijmegen/Nikhef, Nijmegen, The Netherlands; Nikhef National Institute for Subatomic Physics and University of Amsterdam, Amsterdam, The Netherlands; Department of Physics, Northern Illinois University, De Kalb, IL USA; Budker Institute of Nuclear Physics, SB RAS, Novosibirsk, Russia; Department of Physics, New York University, New York, NY USA; Ohio State University, Columbus, OH USA; Faculty of Science, Okayama University, Okayama, Japan; Homer L. Dodge Department of Physics and Astronomy, University of Oklahoma, Norman, OK USA; Department of Physics, Oklahoma State University, Stillwater, OK USA; Palacký University, RCPTM, Olomouc, Czech Republic; Center for High Energy Physics, University of Oregon, Eugene, OR USA; LAL, Université Paris-Sud and CNRS/IN2P3, Orsay, France; Graduate School of Science, Osaka University, Osaka, Japan; Department of Physics, University of Oslo, Oslo, Norway; Department of Physics, Oxford University, Oxford, UK; INFN Sezione di Pavia, Pavia, Italy; Dipartimento di Fisica, Università di Pavia, Pavia, Italy; Department of Physics, University of Pennsylvania, Philadelphia, PA USA; Petersburg Nuclear Physics Institute, Gatchina, Russia; INFN Sezione di Pisa, Pisa, Italy; Dipartimento di Fisica E. Fermi, Università di Pisa, Pisa, Italy; Department of Physics and Astronomy, University of Pittsburgh, Pittsburgh, PA USA; Laboratorio de Instrumentacao e Fisica Experimental de Particulas - LIP, Lisbon, Portugal; Faculdade de Ciências, Universidade de Lisboa, Lisbon, Portugal; Department of Physics, University of Coimbra, Coimbra, Portugal; Centro de Física Nuclear da Universidade de Lisboa, Lisbon, Portugal; Departamento de Fisica, Universidade do Minho, Braga, Portugal; Departamento de Fisica Teorica y del Cosmos and CAFPE, Universidad de Granada, Granada, Spain; Dep Fisica and CEFITEC of Faculdade de Ciencias e Tecnologia, Universidade Nova de Lisboa, Caparica, Portugal; Institute of Physics, Academy of Sciences of the Czech Republic, Prague, Czech Republic; Czech Technical University in Prague, Prague, Czech Republic; Faculty of Mathematics and Physics, Charles University in Prague, Prague, Czech Republic; State Research Center Institute for High Energy Physics, Protvino, Russia; Particle Physics Department, Rutherford Appleton Laboratory, Didcot, UK; Physics Department, University of Regina, Regina, SK Canada; Ritsumeikan University, Kusatsu, Shiga Japan; INFN Sezione di Roma, Rome, Italy; Dipartimento di Fisica, Sapienza Università di Roma, Rome, Italy; INFN Sezione di Roma Tor Vergata, Rome, Italy; Dipartimento di Fisica, Università di Roma Tor Vergata, Rome, Italy; INFN Sezione di Roma Tre, Rome, Italy; Dipartimento di Matematica e Fisica, Università Roma Tre, Rome, Italy; Faculté des Sciences Ain Chock, Réseau Universitaire de Physique des Hautes Energies-Université Hassan II, Casablanca, Morocco; Centre National de l’Energie des Sciences Techniques Nucleaires, Rabat, Morocco; Faculté des Sciences Semlalia, Université Cadi Ayyad, LPHEA-Marrakech, Marrakech, Morocco; Faculté des Sciences, Université Mohamed Premier and LPTPM, Oujda, Morocco; Faculté des Sciences, Université Mohammed V-Agdal, Rabat, Morocco; DSM/IRFU (Institut de Recherches sur les Lois Fondamentales de l’Univers), CEA Saclay (Commissariat à l’Energie Atomique et aux Energies Alternatives), Gif-sur-Yvette, France; Santa Cruz Institute for Particle Physics, University of California Santa Cruz, Santa Cruz, CA USA; Department of Physics, University of Washington, Seattle, WA USA; Department of Physics and Astronomy, University of Sheffield, Sheffield, UK; Department of Physics, Shinshu University, Nagano, Japan; Fachbereich Physik, Universität Siegen, Siegen, Germany; Department of Physics, Simon Fraser University, Burnaby, BC Canada; SLAC National Accelerator Laboratory, Stanford, CA USA; Faculty of Mathematics, Physics and Informatics, Comenius University, Bratislava, Slovak Republic; Department of Subnuclear Physics, Institute of Experimental Physics of the Slovak Academy of Sciences, Kosice, Slovak Republic; Department of Physics, University of Cape Town, Cape Town, South Africa; Department of Physics, University of Johannesburg, Johannesburg, South Africa; School of Physics, University of the Witwatersrand, Johannesburg, South Africa; Department of Physics, Stockholm University, Stockholm, Sweden; The Oskar Klein Centre, Stockholm, Sweden; Physics Department, Royal Institute of Technology, Stockholm, Sweden; Departments of Physics and Astronomy and Chemistry, Stony Brook University, Stony Brook, NY USA; Department of Physics and Astronomy, University of Sussex, Brighton, UK; School of Physics, University of Sydney, Sydney, Australia; Institute of Physics, Academia Sinica, Taipei, Taiwan; Department of Physics, Technion: Israel Institute of Technology, Haifa, Israel; Raymond and Beverly Sackler School of Physics and Astronomy, Tel Aviv University, Tel Aviv, Israel; Department of Physics, Aristotle University of Thessaloniki, Thessaloníki, Greece; International Center for Elementary Particle Physics and Department of Physics, The University of Tokyo, Tokyo, Japan; Graduate School of Science and Technology, Tokyo Metropolitan University, Tokyo, Japan; Department of Physics, Tokyo Institute of Technology, Tokyo, Japan; Department of Physics, University of Toronto, Toronto, ON Canada; TRIUMF, Vancouver, BC Canada; Department of Physics and Astronomy, York University, Toronto, ON Canada; Faculty of Pure and Applied Sciences, University of Tsukuba, Tsukuba, Japan; Department of Physics and Astronomy, Tufts University, Medford, MA USA; Centro de Investigaciones, Universidad Antonio Narino, Bogotá, Colombia; Department of Physics and Astronomy, University of California Irvine, Irvine, CA USA; INFN Gruppo Collegato di Udine, Sezione di Trieste, Udine, Italy; ICTP, Trieste, Italy; Dipartimento di Chimica Fisica e Ambiente, Università di Udine, Udine, Italy; Department of Physics, University of Illinois, Urbana, IL USA; Department of Physics and Astronomy, University of Uppsala, Uppsala, Sweden; Instituto de Física Corpuscular (IFIC) and Departamento de Física Atómica, Molecular y Nuclear and Departamento de Ingeniería Electrónica and Instituto de Microelectrónica de Barcelona (IMB-CNM), University of Valencia and CSIC, Valencia, Spain; Department of Physics, University of British Columbia, Vancouver, BC Canada; Department of Physics and Astronomy, University of Victoria, Victoria, BC Canada; Department of Physics, University of Warwick, Coventry, UK; Waseda University, Tokyo, Japan; Department of Particle Physics, The Weizmann Institute of Science, Rehovot, Israel; Department of Physics, University of Wisconsin, Madison, WI USA; Fakultät für Physik und Astronomie, Julius-Maximilians-Universität, Würzburg, Germany; Fachbereich C Physik, Bergische Universität Wuppertal, Wuppertal, Germany; Department of Physics, Yale University, New Haven, CT USA; Yerevan Physics Institute, Yerevan, Armenia; Centre de Calcul de l’Institut National de Physique Nucléaire et de Physique des Particules (IN2P3), Villeurbanne, France; CERN, 1211 Geneva 23, Switzerland

## Abstract

The paper presents studies of Bose–Einstein Correlations (BEC) for pairs of like-sign charged particles measured in the kinematic range $p_\mathrm{T}>$ 100 MeV and $|\eta |<$ 2.5 in proton collisions at centre-of-mass energies of 0.9 and 7 TeV with the ATLAS detector at the CERN Large Hadron Collider. The integrated luminosities are approximately 7 $\upmu$b$^{-1}$, 190 $\upmu$b$^{-1}$ and 12.4 nb$^{-1}$ for 0.9 TeV, 7 TeV minimum-bias and 7 TeV high-multiplicity data samples, respectively. The multiplicity dependence of the BEC parameters characterizing the correlation strength and the correlation source size are investigated for charged-particle multiplicities of up to 240. A saturation effect in the multiplicity dependence of the correlation source size parameter is observed using the high-multiplicity 7 TeV data sample. The dependence of the BEC parameters on the average transverse momentum of the particle pair is also investigated.

## Introduction

Particle correlations play an important role in the understanding of multiparticle production. Correlations between identical bosons, called Bose–Einstein correlations (BEC), are a well-known phenomenon in high-energy and nuclear physics (for reviews see [1–12]). The BEC are often considered to be the analogue of the Hanbury-Brown and Twiss effect [13–15] in astronomy, describing the interference of incoherently emitted identical bosons [16–19]. They represent a sensitive probe of the space–time geometry of the hadronization region and allow the determination of the size and the shape of the source from which particles are emitted.

The production of identical bosons that are close together in phase space is enhanced by the presence of BEC. The first observation of BEC effects in identically charged pions produced in $p\bar{p}$ collisions was reported in Refs. [20, 21]. Since then, BEC have been studied for systems of two or more identical bosons produced in various types of collisions, from leptonic to hadronic and nuclear collisions (see Refs. [1, 9] and references therein).

Studies of the dependence of BEC on particle multiplicity and transverse momentum are of special interest. They help to understand the multiparticle production mechanism. The size of the source emitting the correlated particles has been observed to increase with particle multiplicity. This can be understood as arising from the increase in the initial geometrical region of overlap of the colliding objects [22]: a large overlap implies a large multiplicity. While this dependence is natural in nucleus–nucleus collisions, the increase of size with multiplicity has also been observed in hadronic and leptonic interactions. In the latter, it is understood as a result of superposition of many sources [8, 23–27] or related to the number of jets [28, 29]. High-multiplicity data in proton–proton interactions can serve as a reference for studies of nucleus–nucleus collisions. The effect is reproduced in both the hydrodynamical/hydrokinetic [30–32] and Pomeron-based [33, 34] approaches for hadronic interactions where high multiplicities play a crucial role. The dependence on the transverse momentum of the emitter particle pair is another important feature of the BEC effect [35]. In nucleus–nucleus collisions the dependence of the particle emitter size on the transverse momentum is explained as a “collective flow”, which generates a characteristic fall-off of the emitter size with increasing transverse momentum [36–38] while strong space–time momentum–energy correlations may offer an explanation in more “elementary” leptonic and hadronic systems [6, 7, 9, 30–32, 35] where BEC measurements serve as a test of different models [30–32, 39–46].

In the present analysis, studies of one-dimensional BEC effects in *pp* collisions at centre-of-mass energies of 0.9 and 7 TeV, using the ATLAS detector [47] at the Large Hadron Collider (LHC), are presented. At the LHC, BEC have been studied by the CMS [48, 49] and ALICE [50, 51] experiments. In the analysis reported here, the studies are extended to the region of high-multiplicities available thanks to the high multiplicity track trigger. The results are compared to measurements at the same or lower energies.

## Analysis

### Two-particle correlation function

Bose–Einstein correlations are measured in terms of a two-particle correlation function,

1 $$\begin{aligned} C_{2} (p_1, p_2) = \frac{\rho (p_1, p_2) }{\rho _0 (p_1, p_2)}\, , \end{aligned}$$

where $p_1$ and $p_2$ are the four-momenta of two identical bosons in the event, $\rho$ is the two-particle density function, and $\rho _0$ is a two-particle density function (known as the reference function) specially constructed to exclude BEC effects. The densities $\rho$ and $\rho _0$ are normalized to unity, i.e. they are the probability density functions.

In order to compare with data over the widest possible range of centre-of-mass energies and system sizes, the density function is parameterized in terms of the Lorentz-invariant four-momentum difference squared, $Q^2$, of the two particles,

2 $$\begin{aligned} Q^2 = - (p_1 - p_2)^2. \end{aligned}$$

The BEC effect is usually described by a function with two parameters: the effective radius parameter *R* and the strength parameter $\lambda$ [52], where the latter is also called the incoherence or chaoticity parameter. A typical functional form is

3 $$\begin{aligned} C_{2}(Q) = \frac{\rho (Q) }{\rho _0 (Q)} = C_{0} [1 + \Omega (\lambda , Q R)] (1 + \varepsilon Q) \, . \end{aligned}$$

In a simplified scheme for fully coherent emission of identical bosons, $\lambda =0$, while for incoherent (chaotic) emission, $\lambda =1$. The *QR* dependence comes from the Fourier transform of the distribution of the space–time points of boson emission. Several different functional forms have been proposed for $\Omega (\lambda ,QR)$. Those used in this paper are described in Sect. 2.4. The fitted parameter $\varepsilon$ takes into account long-distance correlations not fully removed from $\rho _0$. Finally, $C_{0}$ is a normalization constant, typically chosen such that $C_2 (Q)$ is unity for large *Q*. In this paper, the density function $\rho$ is calculated for like-sign charged-particle pairs, with both the $++$ and $--$ combinations included, $\rho (Q) \equiv \rho (++, --)$. All particles are treated as charged pions and no particle identification is attempted. The purity of the analysis sample in terms of identical boson pairs is estimated from MC to be about 70 % (where about 69 % are $\pi ^\pm \pi ^\pm$ and about 1 % are $K^\pm K^\pm$). The effect of the purity is absorbed in the strength parameter $\lambda$, while the results of the analysis on the effective radius parameter *R* were found to be not affected.

### Coulomb correction

The long-range Coulomb force causes a momentum shift between the like-sign and unlike-sign pairs of particles. The density distributions are corrected for this effect by applying the Gamow penetration factor per track pair with a weight 1 / *G*(*Q*) [53–55] (for review see Ref. [82])

4 $$\begin{aligned} \rho _\mathrm{corr} (Q) = {\rho (Q) \over G(Q)} \, , \end{aligned}$$

where the Gamow factor *G*(*Q*) is given by

5 $$\begin{aligned} G(Q) = \frac{2\pi \zeta }{{\mathrm e}^{2\pi \zeta }-1} \end{aligned}$$

with the dimensionless parameter $\zeta$ defined as

6 $$\begin{aligned} \zeta = \pm \frac{\alpha m}{ Q }\, . \end{aligned}$$

Here $\alpha$ is the electromagnetic fine-structure constant and *m* is the pion mass. The sign of $\zeta$ is positive for like-sign pairs and negative for unlike-sign pairs. The resulting correction on $\rho (Q)$ decreases with increasing *Q* and at $Q=0.03$ GeV it is about 20 %. A systematic uncertainty on *G*(*Q*) is considered to cover effects like the extended size of the emission source and other effects, see discussion in Refs. [10, 11]. Neither the Coulomb interaction nor the BEC effect are present in the generation of MC event samples which are used in the analysis. The Coulomb correction is thus not applied to MC events.

### Reference sample

A good choice of the reference sample is important to allow the experimental detection of the BEC signal. Ideally, $\rho _0 (Q)$ should include all momentum correlations except those arising from BEC. Thus, several different choices have been studied to construct an appropriate reference sample.

Most of the proposed approaches use random pairing of particles, such as mixing particles from different events (the “mixed event” technique [56]), or choosing them from the same event but from opposite hemispheres or by rotating the transverse momentum vector of one of the particles of the like-sign pair [9]. Although these mixing techniques reproduce the topology and some properties of the event under consideration and destroy BEC, they violate energy–momentum conservation. Moreover, there are many possible ways to construct the pairs, such as mixing the particles randomly, or keeping some topological constraints such as the event multiplicity, the invariant mass of the pair or the rapidity of the pair. All of these introduce additional biases in the BEC observables. For example, it was observed in dedicated MC studies that the single-ratio correlation functions $C_2$ using reference samples constructed with the event mixing or opposite hemispheres techniques exhibit an increase in the low-*Q* BEC sensitive region. This effect is found to be more pronounced with increase of the multiplicity or average particle-pair transverse momentum and indicates that these reference samples are not suitable.

A natural choice is to use the unlike-sign particle pairs from the same events that are used to form pairs of like-sign particles, i.e., $\rho _0 (Q) \equiv \rho (+-)$, called in the following the unlike-charge reference sample. This sample has the same topology and global properties as the like sign sample $\rho (++,--)$, but is naturally free of any BEC effect. Studying the $C_2$ correlation functions on MC, none of the deficits of the event mixing and opposite hemispheres techniques described above were observed. However, this sample contains hadron pairs from the decay of resonances such as $\rho ,\ \eta , \eta ^{\prime },\ \omega ,\ \phi ,\ K^{*}$, which are not present in the like-sign combinations. These contribute to the low-*Q* region and can give a spurious BEC signature with a large effective radius of the source [57–63].

In this paper, the unlike-charge reference sample is used. To account for the effects of resonances, the two-particle correlation function $C_2(Q)$ is corrected using Monte Carlo simulation without BEC effects via a double-ratio $R_2(Q)$ defined as

7 $$\begin{aligned} R_{2}(Q) = {C_{2}(Q) \over C^{\mathrm MC}_{2}(Q)} = \frac{ \rho {(++,--)} }{ \rho {(+-)}} \Bigg / \frac{ \rho ^{\mathrm MC} {(++,--)} }{ \rho ^{\mathrm MC} {(+-)}}. \end{aligned}$$

### The parameterizations of BEC

Various parameterizations of the $\Omega (\lambda , Q R)$ function can be found in the literature, each assuming a different shape for the particle-emitting source. In the studies presented here, the data are analysed using the following parameterizations:

- the Goldhaber parameterization [20, 21] of a static Gaussian source in the plane-wave approach,
  8 $$\begin{aligned} \Omega = \lambda \cdot \exp {(-R^{2} Q^{2})}, \end{aligned}$$
  which assumes a spherical shape with a radial Gaussian distribution of the emitter;
- the exponential parameterization of a static source
  9 $$\begin{aligned} \Omega = \lambda \cdot \exp {\left( - R Q \right) }, \end{aligned}$$
  which assumes a radial Lorentzian distribution of the source. This parameterization provides a better description of the data at small *Q* values, as discussed in [9].

The first moment of the $\Omega (Q R)$ distribution corresponds to 1 / *R* for the exponential form and to $1/(R\sqrt{\pi })$ for the Gaussian form. To compare the values of the radius parameters obtained from the two functions, the *R* value of the Gaussian should be compared to $R{/}\sqrt{\pi }$ of the exponential form.

## Experimental details

### The ATLAS detector

The ATLAS detector [47] is a multi-purpose particle physics experiment operating at one of the beam interaction points of the LHC. The detector covers almost the whole solid angle around the collision point with layers of tracking detectors, calorimeters and muon chambers. It is designed to study a wide range of physics topics at LHC energies. For the measurements presented in this paper, the tracking devices and the trigger system are of particular importance.

The innermost part of the ATLAS detector is the inner detector (ID), which has full coverage in $\phi$ and covers the pseudorapidity range $|\eta |<2.5$.^1^ It consists of a silicon pixel detector (Pixel), a silicon microstrip detector (SCT) and a transition radiation tracker (TRT). These detectors are immersed in a $\mathrm 2\, T$ solenoidal magnetic field. The Pixel, SCT, and TRT detectors have typical position resolutions of 10, 17 and 130 $\upmu$m for the *r*–$\phi$ coordinate, respectively. In the case of the Pixel and SCT, the resolutions are 115 and 580 $\upmu$m, respectively, for the second measured coordinate. A track from a charged particle traversing the full radial extent of the ID would typically have three Pixel hits, eight or more SCT hits and more than 30 TRT hits.

The ATLAS detector has a three-level trigger system: Level 1 (L1), Level 2 (L2) and event filter (EF). For this measurement, the trigger relies on the L1 signals from the beam pickup timing devices (BPTX) and the minimum-bias (MB) trigger scintillators (MBTS). The BPTX are composed of electrostatic button pick-up detectors attached to the beam pipe and located 175 m from the centre of the ATLAS detector in both directions along the beam pipe. The MBTS are mounted at each end of the detector in front of the liquid-argon end-cap calorimeter cryostats at $z = \pm 3.56$ m. They are segmented into eight sectors in azimuth and two rings in pseudorapidity ($2.09 < |\eta |<2.82$ and $2.82 < |\eta |<3.84$). Data was collected requiring coincidence of BPTX and MBTS signals, where only a single hit in the MBTS was required on either side of the detector. The efficiency of this trigger was studied with events collected with a separate prescaled L1 BPTX trigger, filtered by ID requirements at L2 and at EF level in order to obtain inelastic interactions and found to be 98 % for two selected tracks and 100 % for more than four selected tracks [64, 65].

High-multiplicity track (HM) events were collected at 7 TeV using a dedicated high-multiplicity track trigger. At L1, the collisions were triggered using the summed transverse energy ($\Sigma E_\mathrm{T}$) in all calorimeters, calibrated at the electromagnetic energy scale [66]. The high-multiplicity events were required to have $\Sigma E_\mathrm{T}>20$ GeV. A high number of hits in the SCT was required at L2, while at the EF level at least 124 tracks with $p_\mathrm{T}> 400$ MeV were required to originate from a single vertex.

### Data and Monte Carlo samples

The study is carried out using the *pp*-collision datasets at the centre-of-mass energies $\sqrt{s}=$ 0.9 and 7 TeV that were used in previously published ATLAS studies of minimum-bias interactions [64, 65].

The event and track selection criteria are the same as the ones used for the ATLAS minimum-bias multiplicity analysis [65] with the same minimum-bias trigger and quality criteria for the track reconstruction. All events in these datasets are required to have at least one vertex [67], formed from a minimum of two tracks with $p_\mathrm{T}>$ 100 MeV and consistent with the average beam spot position within the ATLAS detector (primary vertex) [68]. The tracks satisfying the above-mentioned selection criteria are used as the input to determine the corrected distributions, as described in Sect. 3.3. The multiplicity of selected tracks with $p_\mathrm{T}> 100$ MeV and $|\eta |<$ 2.5 within an event is denoted by $n_\mathrm{sel}$.

The contributions from beam–gas collision and from non-collision background (cosmic rays and detector noise) were investigated in Ref. [64] and found to be negligible. Events with more than one primary vertex (less than 0.3 % of the sample) are rejected in order to prevent a bias from multiple proton–proton interactions (pile-up) in the colliding proton bunches.

The same event selection criteria are applied to high-multiplicity events, which are defined to be those with at least 120 selected tracks. To estimate the possible influence of multiple *pp* interactions in the 7 TeV high-multiplicity track trigger data, the distribution of the distances $\Delta z$ between the *z* coordinates of primary and pile-up vertices are studied. The study shows that on average there is less than one pile-up track selected in the HM sample, which has a negligible influence on the BEC studies.

For the measurements at $\sqrt{s}=0.9$ TeV, about $3.6\times 10^{5}$ events with a total of more than $4.5\times 10^{6}$ tracks are after selection, and in the case of $\sqrt{s}=7$ TeV, about $10^{7}$ events with about $2.1\times 10^{8}$ tracks overall are after selection. This corresponds to integrated luminosities of $\sim$7 and $\sim$190 $\upmu$b$^{-1}$ at 0.9 and 7 TeV, respectively. For the measurements at 7 TeV with the high-multiplicity track trigger, about $1.8\times 10^{4}$ events with more than $2.7\times 10^{6}$ tracks overall were after selection. This corresponds to integrated luminosity of $\sim$12.4 nb$^{-1}$.

Large Monte Carlo samples of minimum-bias and high-multiplicity events were generated using the PYTHIA 6.421 Monte Carlo event generator [69] with the ATLAS MC09 set of optimised parameters (tune) [70] ($1.1 \times 10^7$ for $\sqrt{s}=900$ GeV, $2.7 \times 10^7$ for $\sqrt{s}=7$ TeV and $1.8 \times 10^6$ for $\sqrt{s}=7$ TeV high-multiplicity data) with non-diffractive, single-diffractive and double-diffractive processes included in proportion to the cross sections predicted by the model. As discussed in Sect. 2.2, no simulation of the BEC effect is implemented in the generator. This is the baseline Monte Carlo generator which reproduces single-particle spectra [64]. The generated events were passed through the ATLAS simulation and reconstruction chain; the detector simulation program [71] is based on GEANT4 [72]. Dedicated sets of high-multiplicity events were also generated.

For the study of systematic effects, additional Monte Carlo samples were produced using the PHOJET 1.12.1.35 generator [73], PYTHIA with the Perugia0 tune [74]; and the EPOS 1.99_v2965 generator [46] for the high-multiplicity analysis. The PHOJET program uses the dual parton model [75] for low-$p_\mathrm{T}$ physics and is interfaced to PYTHIA for the fragmentation of partons. The EPOS generator is based on an implementation of the QCD-inspired Gribov–Regge field theory describing soft and hard scattering simultaneously, and relies on the same parton distribution functions as used in PYTHIA. The EPOS LHC tune is used with parameters optimised to describe the LHC minimum-bias data [76].

The high-multiplicity PYTHIA MC09 and EPOS samples, each are about two magnitudes larger than the data sample. The $C_2(Q)$ single-ratio correlation functions in MC reproduce data well for $Q> 0.5$ GeV. In the region $Q<0.5$ GeV, the BEC effect is clearly seen in the data $C_2(Q)$ correlation function while no such effect is seen in the MC as expected, since no BEC present in MC.

### Data correction procedure

Following the procedure applied in the previous ATLAS minimum-bias measurements [64, 65], each track is assigned a weight which corrects for the track reconstruction efficiency, for the fraction of secondary particles, for the fraction of the primary particles^2^ outside the kinematic range and for the fraction of fake tracks.^3^ In addition, the effect of events lost due to trigger and vertex reconstruction inefficiencies is corrected for using an event-by-event weight applied to pairs of particles in the *Q* distribution. The efficiency of the high-multiplicity track trigger has been studied in data as a function of the number of reconstructed tracks and is found to be 5 % for 120 selected tracks and to reach a plateau at 100 % once 150 tracks are selected. The measured trigger inefficiency is used to correct the experimental distributions and is found to have negligible impact on the extraction of the BEC parameters discussed in Sect. 5.

The multiplicity distributions are corrected to the particle level using an iterative method that follows the Bayesian approach [77] as it is described in Refs. [64, 65]. An unfolding matrix reflecting the probability of reconstructing $n_\mathrm{sel}$ charged tracks in an event with generated charged-particle multiplicity $n_\mathrm{ch}$ is populated using Monte Carlo simulation and applied to the data. The unfolding matrix is built using the ATLAS MC09 PYTHIA tune [70]. The unfolding procedure converges after the fifth iteration. It is found that the corrected multiplicity distribution agrees well with the published result [64, 65]. The unfolding procedure of the 7 TeV high-multiplicity data follows the same technique and unfolding matrix used in the previous analysis of minimum-bias data in Ref. [64], restricted to the region of high charged particle multiplicity specific to this analysis, and convolved with a normalised Gaussian distribution to account for the experimental resolution on the number of selected tracks. It is found that a number of 120 selected tracks at detector level, $n_\mathrm{sel}$, corresponds to about 150 charged particle, $n_\mathrm{ch}$, at particle level. Momentum distributions are unfolded in a similar way.

For all distributions, closure tests are carried out using Monte Carlo samples corrected according to the same procedure as used in the data. The difference obtained between the reweighted distributions and those at the particle level is due to tracking effects such as a smaller reconstruction efficiency for pairs of tracks with very small opening angle. These effects are small for correlation functions constructed using data, typically 1–3 %, and are included in the systematic uncertainty. In the case of the unfolded *Q* distributions, the data are corrected for the bias from secondary tracks using Monte Carlo simulation and the corresponding systematic uncertainty is obtained by variation of the amount of material in the inner detector by $\pm 10$ %.

## Systematic uncertainties

The systematic uncertainties of the inclusive fit parameters, *R* and $\lambda$, of the exponential model are summarized in Table 1. The following contributions to the systematic uncertainties on the fitted parameters are considered.

The systematic uncertainties resulting from the track reconstruction efficiency, which are parameterized in bins of $p_\mathrm{T}$ and $\eta$, were determined in earlier analyses [64, 65]. These cause uncertainties in the track weights of particle pairs in the *Q* distributions entering the correlation functions.

The effects of track splitting and merging are sizeable only for very low *Q* values (smaller than 5 MeV), and are found to be negligible for the measurements with $Q \ge 20$ MeV.

The leading source of systematic uncertainty is due to differences in the Monte Carlo generators used to calculate the $R_2$ correlation function from the $C_2$ correlation function. The corresponding contribution to the systematic uncertainty is estimated as the root-mean-squared (RMS) spread of the results obtained for the different Monte Carlo datasets. The statistical uncertainties arising from the Monte Carlo datasets are negligibly small.

The systematic uncertainty due to Coulomb corrections is estimated by varying the corrections by $\pm 20$ %.

The influence of the fit range is estimated by changing the upper bound of the *Q* range from the nominal 2 GeV: decreasing it to 1.5 GeV and increasing it up to 2.5 GeV. The latter better estimates the uncertainty due the long-range correlations. This contribution is taken into account by the value of $\varepsilon$, the parameter in the linear term of Eq. (3) describing the long-range correlations.

Other effects contributing to the systematic uncertainties are the lowest value of *Q* for the fit, the bin size and exclusion of the interval $0.5\le Q \le 0.9$ GeV due to the overestimate of the $\rho$ meson contribution in the Monte Carlo simulations, as discussed in the following Sect. 5.1. These uncertainties are estimated by varying the lowest *Q* value in the fit by $\pm 10$ MeV, by changing the bin size by $\pm 10$ MeV, and by broadening the excluded interval by 100 MeV on both sides.

The background of photon conversions into $e^+e^-$ pairs was studied and found to be negligible.

To test the effect of treating all charged particles as pions, the double-ratio correlation functions $R_2$ are also obtained using only identical particles in the Monte Carlo sample to compute the correction. The resulting BEC parameters fitted to the $R_2$ functions defined this way show negligible differences to the nominal result and no further systematic uncertainties are assigned.

Finally, the systematic uncertainties are combined by adding them in quadrature and the resulting values are given in the bottom row of Table 1.

The same sources of uncertainty are considered for the differential measurements in $n_\mathrm{ch}$ and the average transverse momentum $k_\mathrm{T}$ of a pair, and their impact on the fit parameters is found to be similar in size.

**Table 1:** Systematic uncertainties on $\lambda$ and *R* for the exponential fit of the two-particle double-ratio correlation function $R_2 (Q)$ in the full kinematic region at $\sqrt{s}=$0.9 and 7 TeV for minimum-bias and high-multiplicity (HM) events

	0.9 TeV		7 TeV		7 TeV (HM)	
Source	$\lambda$ (%)	*R* (%)	$\lambda$ (%)	*R* (%)	$\lambda$ (%)	*R* (%)
Track reconstruction efficiency	0.6	0.7	0.3	0.2	1.3	0.3
Track splitting and merging	Negligible	Negligible	Negligible	Negligible	Negligible	Negligible
Monte Carlo samples	14.5	12.9	7.6	10.4	5.1	8.4
Coulomb correction	2.6	0.1	5.5	0.1	3.7	0.5
Fitted range of *Q*	1.0	1.6	1.6	2.2	5.5	6.0
Starting value of *Q*	0.4	0.3	0.9	0.6	0.5	0.3
Bin size	0.2	0.2	0.9	0.5	4.1	3.4
Exclusion interval	0.2	0.2	1	0.6	0.7	1.1
Total	14.8	13.0	9.6	10.7	9.4	10.9

## Results

### Two-particle correlations

**Fig. 1:**
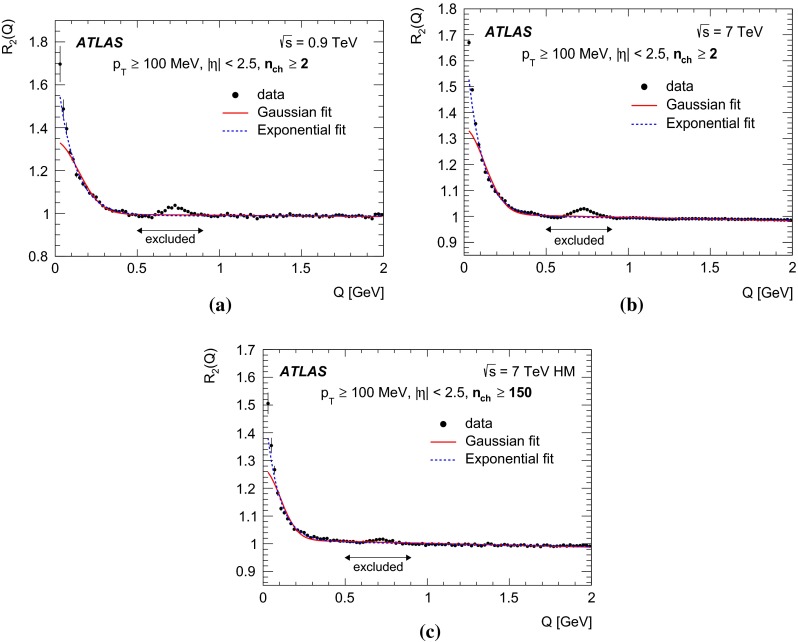
The two-particle double-ratio correlation function $R_2 (Q)$ for charged particles in *pp* collisions at **a** $\sqrt{s}=$0.9 TeV, **b** 7 TeV and **c** 7 TeV high-multiplicity events. The *lines* show the Gaussian and exponential fits as described in the legend. The region excluded from the fits is indicated. The *error bars* represent the statistical uncertainties

In Fig. 1 the double-ratio $R_2(Q)$ distributions, measured for 0.9 and 7 TeV, are compared with Gaussian and exponential fitting functions, Eqs. (8) and (9). The fits are performed in the *Q* range 0.02–2 GeV and with a bin width of 0.02 GeV. The upper *Q* limit is chosen to be far away from the low-*Q* region, which is sensitive to BEC effects and resonances. Around $Q\sim 0.7$ GeV  there is a visible bump which is due to an overestimate of $\rho \rightarrow \pi ^+ \pi ^-$ decays in the Monte Carlo simulation. Therefore the region $0.5 \le Q \le 0.9$ GeV is excluded from the fits. As seen in Fig. 1, the Gaussian function does not describe the low-*Q* region while the exponential function provides a good description of the data.

The resolution of the *Q* variable is better than 10 MeV for the region most sensitive to BEC effect, $Q < 0.4$ GeV. The *Q* resolution is included in the fit of $R_2$ by convolving the fitting function with a Gaussian detector resolution function. The change in the fit results from those with no convolution applied is found to be negligible.

In the process of fitting $R_2(Q)$ with the exponential function, large $\chi ^2$ values are observed, in particular for the 7 TeV sample where statistical uncertainties on the fitted data points are below 2–4 %. These large $\chi ^2$ values can be traced back to a small number of individual points or small cluster of points. The removal of these points does not change the results of the fit while the $\chi ^2$ substantially improves. In the analysis of the 7 TeV data, for most of the considered cases, the expected statistical uncertainties are small compared to the systematic ones, therefore only total uncertainties on the fitted parameters are given. The latter include the statistical uncertainties rescaled by $\sqrt{\chi ^2/\mathrm {ndf}}$ [78]. For consistency, the same treatment is applied to the 0.9 TeV analysis where the statistical uncertainties are of the same order of magnitude as the systematic ones.

**Fig. 2:**
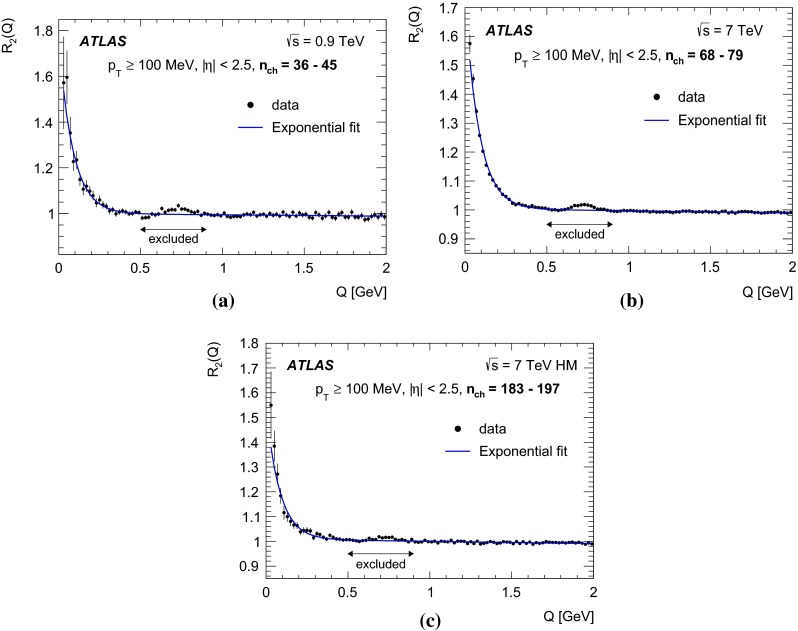
The two-particle double-ratio correlation function $R_2 (Q)$ for charged particles in *pp* collisions for multiplicity intervals: **a** $36 \le n_\mathrm{ch} < 45$ at $\sqrt{s}=$0.9 TeV, **b** $68 \le n_\mathrm{ch} < 79$ at 7 TeV and **c** $183 \le n_\mathrm{ch} < 197$ at 7 TeV high-multiplicity events. The *lines* show the results of the exponential fit. The region excluded from the fits is indicated. The *error bars* represent the statistical uncertainties

The results of BEC parameters for exponential fits of the two-particle double-ratio correlation function $R_2 (Q)$ for events with the unlike-charge reference sample are

$$\begin{aligned}&\lambda = 0.74\pm 0.11,\, R = (1.83\pm 0.25)\ \mathrm{fm}\,\, \mathrm{at}\, \sqrt{s} = 0.9\,\mathrm{TeV}\, \mathrm{for}\, n_\mathrm{ch} \ge 2,\\&\lambda = 0.71\pm 0.07,\, R = (2.06\pm 0.22)\ \mathrm{fm}\, \mathrm{at}\, \sqrt{s} = 7\,\mathrm{TeV}\, \mathrm{for}\, n_\mathrm{ch} \ge 2,\\&\lambda = 0.52\pm 0.06,\, R = (2.36\pm 0.30)\ \mathrm{fm}\, \mathrm{at}\, \sqrt{s} = 7\,\mathrm{TeV}\, \mathrm{for}\, n_\mathrm{ch} \ge 150. \end{aligned}$$

The values of the fitted parameters are close to the values obtained by the CMS [49] and ALICE [50] experiments.

### Multiplicity dependence

The $R_2(Q)$ functions defined in Eq. (7), are shown for various multiplicity intervals in Fig. 2 for 0.9, 7 and 7 TeV high-multiplicity data. The multiplicity intervals are chosen so as to be similarly populated and comparable to those used by other LHC experiments [48–51]. Only the exponential fit is shown. As in the fit procedure for the inclusive case, the detector *Q* resolution is included in the fits.

**Fig. 3:**
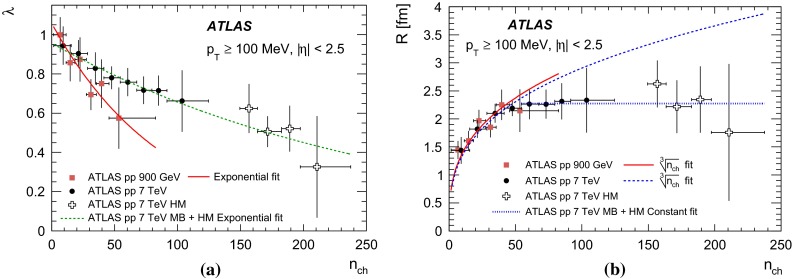
Multiplicity, $n_\mathrm {ch}$, dependence of the parameters: **a** $\lambda$ and **b** *R* obtained from the exponential fit to the two-particle double-ratio correlation functions $R_2 (Q)$ at $\sqrt{s}=$0.9 and 7 TeV. The *solid* and *dashed curves* are the results of **a** the exponential and **b** $\root 3 \of {n_\mathrm{ch} }$ for $n_\mathrm{ch} < 55$ fits. The *dotted line* in **b** is a result of a constant fit to minimum-bias and high-multiplicity events data at 7 TeV for $n_\mathrm{ch} \ge 55$. The *error bars* represent the quadratic sum of the statistical and systematic uncertainties

Within the multiplicity studies, the BEC parameters are also measured by excluding the low-multiplicity events, $n_\mathrm{ch}< 8$, expected to be contaminated by diffractive physics [64]. No noticeable changes in the strength and radius parameters for $n_\mathrm{ch} \ge 8$ are observed compared to the full multiplicity range for $n_\mathrm{ch} \ge 2$.

The multiplicity dependence of the $\lambda$ and *R* parameters is shown in Fig. 3. The $\lambda$ parameter decreases with multiplicity, faster for 0.9 TeV than for 7 TeV interactions. The decrease of the $\lambda$ parameter with $n_\mathrm{ch}$ is found to be well fitted with the exponential function $\lambda (n_\mathrm{ch}) = \gamma \, {\mathrm e}^{- \delta n_\mathrm{ch}}$. The fit parameter values are presented in Table 2 for 0.9 TeV and for the combined nominal and high-multiplicity 7 TeV data.

The *R* parameter increases with multiplicity up to about $n_\mathrm{ch} \simeq 50$ independently of the center of mass energy. For higher multiplicities, the measured *R* parameter is observed to be independent of multiplicity. For $n_\mathrm{ch} \le 82$ at 0.9 TeV and $n_\mathrm{ch} < 55$ at 7 TeV the $n_\mathrm{ch}$ dependence of *R* is fitted with the function $R (n_\mathrm{ch}) = \alpha \root 3 \of {n_\mathrm{ch} }$, similar to that used in heavy-ion studies [5, 51]. The results of the fit are presented in Table 2 and are close to the CMS results [49]. The fit parameters do not change significantly within uncertainties if data points with $n_\mathrm{ch}>55$ are included in the fit, while the quality of the fit significantly degrades. Therefore the fit is limited to the data points with $n_\mathrm{ch}\le 55$. The $n_\mathrm{ch}$ dependence of *R* at 7 TeV is fitted with a constant $R (n_\mathrm{ch}) = \beta$ for $n_\mathrm{ch}> 55$; the resulting value is given in Table 2. Qualitatively CMS [49] and UA1 [79] results for the radius parameter follow the same trend as a function of $n_\mathrm{ch}$ as ATLAS data points up to $n_\mathrm{ch} \le 55$. The ATLAS and ALICE [50, 51] results on the multiplicity dependence of the radius parameter cannot be directly compared due to much narrower $\eta$ region used by ALICE.

The observed change of the fitted parameters with multiplicity has been predicted in Refs. [9, 23–27], and is similar to the one also observed in $e^+ e^-$ interactions [28], however the saturation of *R* for very high multiplicity is observed for the first time.

The saturation of *R* at high multiplicities is expected in a Pomeron-based model [33, 34] as the consequence of the overlap of colliding protons, with the value of the radius parameter at $n_\mathrm{ch}\approx 70$ close to the one obtained in the present studies. However, the same model predicts that above $n_\mathrm{ch}\approx 70$, *R* will decrease with multiplicity, returning to its low-multiplicity value which is not supported by the data.

### Dependence on the transverse momentum of the particle pair

The average transverse momentum $k_\mathrm{T}$ of a particle pair is defined as half of the magnitude of the vector sum of the two transverse momenta, $k_\mathrm{T}= |\mathbf{p}_{\mathrm{T}, 1} + \mathbf{p}_{\mathrm{T}, 2}|/2$. The study is performed in the $k_\mathrm{T}$ intervals which are chosen in a way to be similarly populated and, as for the multiplicity bins, to be similar to the intervals used by other LHC experiments [48–51].

**Fig. 4:**
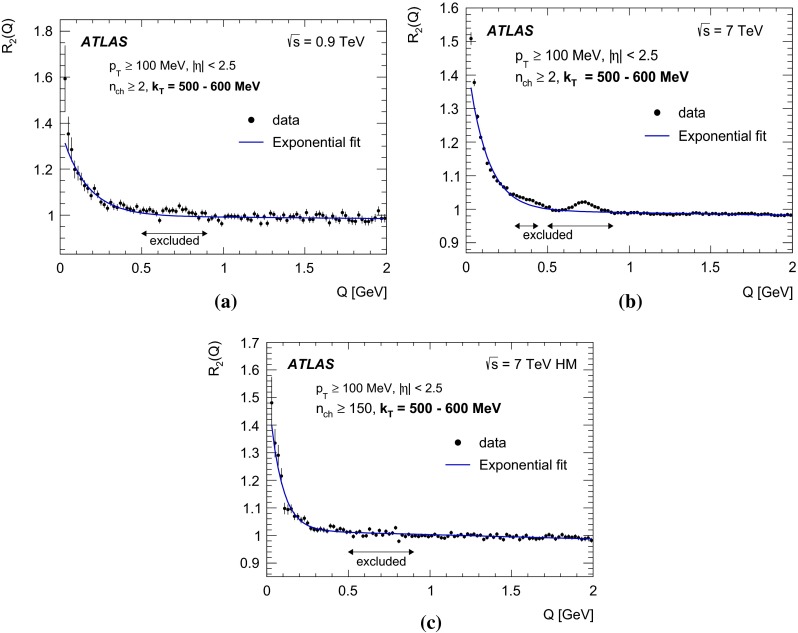
The two-particle double-ratio correlation function $R_2 (Q)$ for charged particles in *pp* collisions for $500\le k_\mathrm{T}< 600$ MeV interval at **a** $\sqrt{s}=$0.9 TeV, **b** 7 TeV and **c** 7 TeV high-multiplicity events. The average transverse momentum $k_\mathrm{T}$ of the particle pairs is defined as $k_\mathrm{T}= |\mathbf{p}_{\mathrm{T}, 1} + \mathbf{p}_{\mathrm{T}, 2}|/2$. The *lines* show the exponential fits. The region excluded from the fits is indicated. The *error bars* represent the statistical uncertainties

As an example, the $R_2(Q)$ distributions for the $500\le k_\mathrm{T}\le 600$ MeV interval for the 0.9, 7 TeV and high-multiplicity 7 TeV samples are shown in Fig. 4 together with the results of the corresponding exponential fit. For the $R_2 (Q)$ correlation function measured at 7 TeV (see Fig. 4b), there is an indication that the Monte Carlo simulation overestimates the production and decay of the $\omega$-meson in the *Q* region of 0.3–0.44 GeV. This region is thus excluded from the fit range for $k_\mathrm{T}>500$ MeV bin results.

In the region most important for the BEC parameters, the quality of the exponential fit is found to deteriorate as $k_\mathrm{T}$ increases. This is due to the fact that at large $k_\mathrm{T}$ values, the characteristic BEC peak becomes steeper than the exponential function can accommodate. Despite the deteriorating fit quality, the behaviour of the fitted parameters is presented for comparison with previous experiments.

**Table 2:** Results of fitting the multiplicity, $n_\mathrm {ch}$, and the transverse momentum of the pair, $k_\mathrm {T}$, dependence of the BEC parameters *R* and $\lambda$ with different functional forms and for different data samples. The error represent the quadratic sum of the statistical and systematic uncertainties

BEC param.	Fit function	0.9 TeV	7 TeV	
			Minimum-bias events	High-multiplicity events
$R(n_\mathrm{ch})$	$\alpha \root 3 \of {n_\mathrm{ch} }$	$\alpha = 0.64\pm 0.07$ fm ($n_\mathrm{ch} \le 82$)	$\alpha = 0.63\pm 0.05$ fm ($n_\mathrm{ch} < 55$)	–
	$\beta$	–	$\beta = 2.28\pm 0.32$ fm ($n_\mathrm{ch} \ge 55$)	
$\lambda (n_\mathrm{ch})$	$\gamma \, {\mathrm e}^{- \delta n_\mathrm{ch}}$	$\gamma = 1.06\pm 0.10$	$\gamma =0.96\pm 0.07$	
		$\delta = 0.011\pm 0.004$	$\delta =0.0038\pm 0.0008$	
$R(k_\mathrm{T})$	$\xi \, {\mathrm e}^{- \kappa k_\mathrm{T}}$	$\xi = 2.64\pm 0.33$ fm	$\xi = 2.88\pm 0.27$ fm	$\xi = 3.39\pm 0.54$ fm
		$\kappa = 1.48\pm 0.67$ GeV$^{-1}$	$\kappa = 1.05\pm 0.58$ GeV$^{-1}$	$\kappa = 0.92\pm 0.73$ GeV$^{-1}$
$\lambda (k_\mathrm{T})$	$\mu \, {\mathrm e}^{- \nu k_\mathrm{T}}$	$\mu = 1.20\pm 0.18$	$\mu = 1.12\pm 0.10$	$\mu = 0.75\pm 0.10$
		$\nu = 2.00\pm 0.35$ GeV$^{-1}$	$\nu = 1.54\pm 0.26$ GeV$^{-1}$	$\nu = 0.91\pm 0.45$ GeV$^{-1}$

**Fig. 5:**
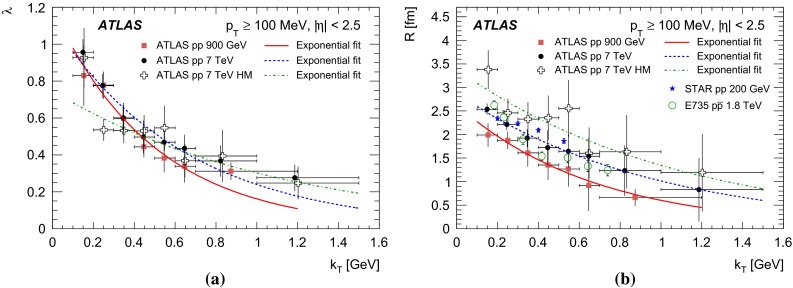
The $k_\mathrm{T}$ dependence of the fitted parameters: **a** $\lambda$ and **b** *R* obtained from the exponential fit to two-particle double-ratio at $\sqrt{s}=$0.9, 7 and 7 TeV high-multiplicity events. The average transverse momentum $k_\mathrm{T}$ of the particle pairs is defined as $k_\mathrm{T}= |\mathbf{p}_{\mathrm{T}, 1} + \mathbf{p}_{\mathrm{T}, 2}|/2$. The *solid*, *dashed* and *dash-dotted curves* are results of the exponential fits for 0.9, 7 and 7 TeV high-multiplicity data, respectively. The results are compared to the corresponding measurements by the E735 experiment at the Tevatron [80], and by the STAR experiment at RHIC [81]. The *error bars* represent the quadratic sum of the statistical and systematic uncertainties

The fit values of the $\lambda$ and *R* parameters are shown in Fig. 5 as a function of $k_\mathrm {T}$. The values of both $\lambda$ and *R* decrease with increasing $k_\mathrm{T}$.

The decrease of $\lambda$ with $k_\mathrm{T}$ is well described by an exponential function, $\lambda (k_\mathrm{T}) = \mu \, {\mathrm e}^{- \nu k_\mathrm{T}}$. The $k_\mathrm{T}$ dependence of the *R* parameter is also found to follow an exponential decrease, $R (k_\mathrm{T}) = \xi \, {\mathrm e}^{- \kappa k_\mathrm{T}}$. The shapes of the $k_\mathrm{T}$ dependence are similar for the 7 TeV and the 7 TeV high-multiplicity data. The results of the fits are presented in Table 2.

In Fig. 5b, the $k_\mathrm{T}$ dependence of the *R* parameter is compared to the measurements performed by the E735 [80] and the STAR [81] experiments with mixed-event reference samples. These earlier results were obtained from Gaussian fits to the single-ratio correlation functions and therefore the values of the measured radius parameters are multiplied by $\sqrt{\pi }$ as discussed in Sect. 2.4. The values of the parameters are observed to be energy-independent within the uncertainties.

**Fig. 6:**
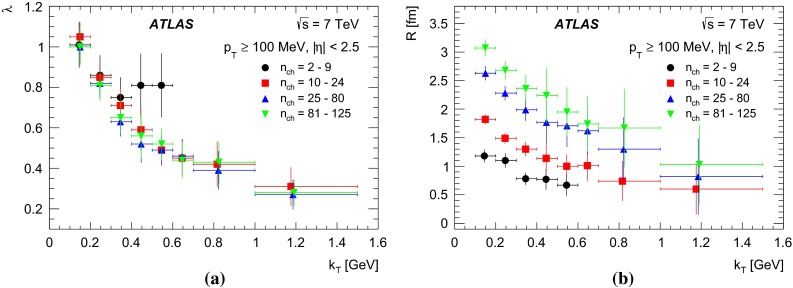
The $k_\mathrm{T}$ dependence of the fitted parameters: **a** $\lambda$ and **b** *R* obtained from the exponential fit to the two-particle double-ratio correlation function $R_2 (Q)$ at $\sqrt{s}=$ 7 TeV for the different multiplicity regions: $2\le n_\mathrm{ch} \le 9$ (*circles*), $10 \le n_\mathrm{ch} \le 24$ (*squares*), $25 \le n_\mathrm{ch} \le 80$ (*triangles*) and $81 \le n_\mathrm{ch} \le 125$ (*inverted triangles*). The average transverse momentum $k_\mathrm{T}$ of the particle pairs is defined as $k_\mathrm{T}= |\mathbf{p}_{\mathrm{T}, 1} + \mathbf{p}_{\mathrm{T}, 2}|/2$. The *error bars* represent the quadratic sum of the statistical and systematic uncertainties

In Fig. 6, the $k_\mathrm{T}$ dependence of $\lambda$ and *R*, obtained for the 7 TeV data, is also studied in various multiplicity regions: $2 \le n_\mathrm{ch} \le 9$; $10 \le n_\mathrm{ch} \le 24$; $25 \le n_\mathrm{ch} \le 80$; and $81 \le n_\mathrm{ch} \le 125$. The decrease of $\lambda$ with $k_\mathrm{T}$ is nearly independent of multiplicity for $n_\mathrm{ch} > 9$ and the same as for the inclusive case. For $n_\mathrm{ch} \le 9$ no conclusions can be drawn due to the large uncertainties. The *R*-parameter decreases with $k_\mathrm{T}$ and exhibits an increase with increasing multiplicity as was observed for the fully inclusive case.

## Summary and conclusions

The two-particle Bose–Einstein correlations of like-sign hadrons with $p_\mathrm{T}>$ 100 MeV and $|\eta |<$ 2.5 produced in *pp* collisions recorded by the ATLAS detector at 0.9 and 7 TeV at the CERN LHC are studied. In addition to minimum-bias data, high-multiplicity data recorded at 7 TeV using a dedicated trigger are investigated. The integrated luminosities are about 7 $\upmu$b$^{-1}$, 190 $\upmu$b$^{-1}$ and 12.4 nb$^{-1}$ for 0.9, 7 TeV minimum-bias and 7 TeV high-multiplicity data samples, respectively.

The studies were performed using the double-ratio correlation function. In the double-ratio method, the single-ratio correlation function obtained from the data is divided by a similar single-ratio calculated using Monte Carlo events, which do not have BEC effects. The reference sample for each of the two single-ratios is constructed from unlike-sign charged-particle pairs.

A clear signal of Bose–Einstein correlations is observed in the region of small four-momentum difference. To quantitatively characterize the BEC effect, Gaussian and exponential parametrizations are fit to the measured correlation functions. As observed in studies performed by other experiments, the Gaussian parameterization provides a poor description of the BEC-enhanced region and hence the exponential parameterization is used for the final results.

The BEC parameters are studied as a function of the charged-particle multiplicity and the transverse momentum of the particle pair. A decrease of the correlation strength $\lambda$ along with an increase of the correlation source size parameter *R* are found with increasing charged-particle multiplicity. On the other hand no dependence of *R* on the centre-of-mass energy of *pp* collisions is observed. For the first time a saturation of the source size parameter is observed for multiplicity $n_\mathrm{ch}\ge 55$. The correlation strength $\lambda$ and the source size parameter *R* are found to decrease with increasing average transverse momentum of a pair. The study of BEC in ($n_\mathrm{ch}, k_\mathrm{T}$) bins at 7 TeV shows a decrease of the *R* parameter with $k_\mathrm{T}$ for different multiplicity ranges, while the *R* values increase with multiplicity. The $\lambda$ parameter is found to decrease with $k_\mathrm{T}$ independently of the multiplicity range. These resemble the dependences for the inclusive case at 7 TeV for minimum-bias and high-multiplicity data.

A comparison is made to the measurements by other experiments at the same and lower energies where possible. The measurements presented here complement the earlier measurements by extending the studies to higher multiplicities and transverse momenta. This has allowed a first observation of a saturation in the magnitude of the source radius parameter at high charged-particle multiplicities, and confirms the exponential decrease, observed in previous measurements of the radius parameters with increasing pair transverse momenta.

## Footnotes

1 ATLAS uses a right-handed coordinate system with its origin at the nominal interaction point (IP) in the centre of the detector and the *z*-axis along the beam pipe. The *x*-axis points from the IP to the centre of the LHC ring, and the *y*-axis points upward. Cylindrical coordinates $(r,\phi )$ are used in the transverse plane, $\phi$ being the azimuthal angle around the beam pipe. The pseudorapidity is defined in terms of the polar angle $\theta$ as $\eta =-\ln \tan (\theta /2)$.

2 In the Monte Carlo simulations, primary charged particles are defined as charged particles with a mean lifetime $\tau > 0.3 \times 10^{-10}$ s either directly produced in *pp* collisions or from the subsequent decay of particles with a shorter lifetime.

3 Fake tracks are tracks constructed from tracker noise and/or hits which are not produced by a single-particle.

## References

[1] W.A. Zajc, in *Hadronic Multiparticle Production (Advanced Series on Directions in High Energy Physics)*, vol. 2. ed. by P. Carruthers (World Scientific, Singapore, 1988), p. 235

[2] Kanki T, Kinoshita K, Sumiyoshi H, Takagi F (1988). Prog. Theor. Phys. Suppl..

[3] Kanki T, Kinoshita K, Sumiyoshi H, Takagi F (1989). Prog. Theor. Phys. Suppl..

[4] Boal DH, Gelbke CK, Jennings BK (1990). Rev. Mod. Phys..

[5] M.A. Lisa, S. Pratt, R. Soltz, U. Wiedemann, Ann. Rev. Nucl. Part. Sci. **55**, 357 (2005). arXiv:nucl-ex/0505014

[6] Kittel W (2001). Acta Phys. Polon. B.

[7] Csörgő T (2002). Heavy Ion Phys..

[8] Alexander G (2003). Rep. Prog. Phys..

[9] Kittel W, De Wolf EA (2005). Soft Multihadron Dynamics.

[10] R.M. Weiner, *Introduction to Bose–Einstein Correlations and Subatomic Interferometry* (Wiley, Chichester, 2000)

[11] R.M. Weiner, Phys. Rep. **327**, 249 (2000). arXiv:hep-ph/9904389

[12] Suzuki N, Biyajima M (1999). Phys. Rev. C.

[13] R. Hanbury Brown, R.Q. Twiss. Phil. Mag. **45**, 663 (1954)

[14] R. Hanbury Brown, R.Q. Twiss, Nature **177**, 27 (1956)

[15] R. Hanbury Brown, R.Q. Twiss, Nature **178**, 1046 (1956)

[16] Kopylov GI, Podgoretskiĭ MI (1972). Sov. J. Nucl. Phys..

[17] Kopylov GI, Podgoretskiĭ MI (1974). Sov. J. Nucl Phys..

[18] Shuryak EV (1973). Phys. Lett. B.

[19] Cocconi G (1974). Phys. Lett. B.

[20] Goldhaber G (1959). Phys. Rev. Lett..

[21] Goldhaber G (1960). Phys. Rev..

[22] Barshay S (1983). Phys. Lett. B.

[23] Suzuki N, Biyajima M (1999). Phys. Rev. C.

[24] B. Buschbeck, H.C. Eggers, P. Lipa, Phys. Lett. B **481**, 187 (2000). arXiv:hep-ex/0003029

[25] B. Buschbeck, H.C. Eggers, P. Lipa, Nucl. Phys. B, Proc. Suppl. **92**, 235 (2001). arXiv:hep-ph/0011292

[26] Alexander G, Sarkisyan EKG (2000). Phys. Lett. B.

[27] G. Alexander, E.K.G. Sarkisyan, Nucl. Phys. B Proc. Suppl. **92**, 211 (2001). arXiv:hep-ph/0008174

[28] G. Alexander et al., OPAL Collaboration, Z. Phys. C **72**, 389 (1996)

[29] Alexander G (2012). J. Phys. G.

[30] Sinyukov Yu.M., Shapoval VM (2013). Phys. Rev. D.

[31] V.M. Shapoval, P. Braun-Munzinger, Iu.A. Karpenko, Yu.M. Sinyukov, Phys. Lett. B **725**, 139 (2013).arXiv:1304.3815

[32] Yu.M. Sinyukov, S.V. Akkelin, Iu.A. Karpenko, V.M. Shapoval, Adv. High Energy Phys. **2013**, 198928 (2013)

[33] Schegelsky VA, Martin AD, Ryskin MG, Khoze VA (2011). Phys. Lett. B.

[34] Ryskin MG, Schegelsky VA (2011). Nucl. Phys. Proc. Suppl..

[35] E. Shuryak, arXiv:1412.8393

[36] Pratt S (1984). Phys. Rev. Lett..

[37] Makhlin AN, Sinyukov YM (1988). Z. Phys. C.

[38] Hama Y, Padula SS (1988). Phys. Rev. D.

[39] Csörgő T, Zimányi J (1990). Nucl. Phys. A.

[40] Lörstad B, Sinyukov YuM (1991). Phys. Lett. B.

[41] Csörgő T, Kittel W, Metzger WJ, Novák T (2008). Phys. Lett. B.

[42] P. Bożek, Acta Phys. Pol. B **41**, 837 (2010). arXiv:0911.2392

[43] Nilsson MS, Bravina LV, Zabrodin EE, Malinina LV, Bleibel J (2011). Phys. Rev. D.

[44] Li Q, Graef G, Bleicher M (2013). J. Phys. Conf. Ser..

[45] G. Alexander, I. Ben Mordechai, J. Phys. G **40**, 125101 (2013)

[46] Werner K, Karpenko I, Pierog T, Mikhailov K (2011). Phys. Rev. C.

[47] ATLAS Collaboration, JINST **3**, S08003 (2008)

[48] CMS Collaboration, Phys. Rev. Lett. **105**, 032001 (2010). arXiv:1005.3294

[49] CMS Collaboration, J. High Energy Phys. **05**, 029 (2011). arXiv:1101.3518

[50] K. Aamodt et al., ALICE Collaboration, Phys. Rev. D **82**, 052001 (2010). arXiv:1007.0516

[51] K. Aamodt et al., ALICE Collaboration, Phys. Rev. D **84**, 112004 (2011). arXiv:1101.3665

[52] Deutschmann M (1982). Nucl. Phys. B.

[53] Gyulassy M, Kauffmann SK, Wilson LW (1979). Phys. Rev. C.

[54] Pratt S (1986). Phys. Rev. D.

[55] I. Juricic et al., Phys. Rev. D **39**, 1 (1989) 10.1103/physrevd.39.1 9959471

[56] Kopylov GI (1974). Phys. Lett. B.

[57] Grassberger P (1977). Nucl. Phys. B.

[58] Gyulassy M, Padula SS (1989). Phys. Lett. B.

[59] Padula SS, Gyulassy M (1989). Nucl. Phys. A.

[60] Kulka K, Lörstad B (1990). Z. Phys. C.

[61] Bowler MG (1990). Z. Phys. C.

[62] Lednický R, Progulova TB (1992). Z. Phys. C.

[63] Padula SS, Gyulassy M (1992). Nucl. Phys. A.

[64] ATLAS Collaboration, N. J. Phys. **13**, 053033 (2011). arXiv:1012.5104

[65] ATLAS Collaboration, Phys. Lett. B **688**, 21 (2010). arXiv:1003.3124

[66] ATLAS Collaboration, ATLAS-LARG-PUB-2008-002 (2008)

[67] Piacquadio G, Prokofiev K, Wildauer A (2008). J. Phys. Conf. Ser..

[68] ATLAS Collaboration, ATLAS-CONF-2010-027 (2010)

[69] Sjöstrand T, Mrenna S, Skands PZ (2006). J. High Energy Phys..

[70] ATLAS Collaboration, ATL-PHYS-PUB-2010-002 (2010)

[71] ATLAS Collaboration, Eur. Phys. J. C **70**, 823 (2010). arXiv:1005.4568

[72] S. Agostinelli et al., GEANT4 Collaboration, Nucl. Instrum. Methods A **506**, 250 (2002)

[73] R. Engel et al., Z. Phys. C **66**, 203 (1995)

[74] P.Z. Skands, *The Perugia Tune*. arXiv:0905.3418

[75] Capella A (1994). Phys. Rep..

[76] ATLAS Collaboration, J. High Energy Phys. **11**, 033 (2012). arXiv:1208.6256

[77] G. D’ Agostini, Nucl. Instr. Meth. A **362**, 487 (1995)

[78] K.A. Olive et al., Particle Data Group, Chin. Phys. C **38**, 090001 (2014)

[79] C. Albajar et al., UA1 Collaboration, Phys. Lett. B **226**, 410 (1989)

[80] Alexopoulos T (1993). Phys. Rev. D.

[81] M.M. Aggarwal et al., STAR Collaboration, Phys. Rev. C **83**, 064905 (2011). arXiv:1004.0925

[82] B. Tomášik, U.A. Wiedemann, in *Quark–Gluon Plasma*, vol. 3, ed. by R.C. Hwa, X.-N. Wang (World Scientific, Singapore, 2004), p. 715. hep-ph/0210250

